# Evaluation of digital economy development level based on multi-attribute decision theory

**DOI:** 10.1371/journal.pone.0270859

**Published:** 2022-10-20

**Authors:** Jinqi Su, Ke Su, Shubin Wang

**Affiliations:** 1 School of Economics and Management, Xi’an University of Posts and Telecommunications, Shaanxi, Xi’an, China; 2 Research Center of Chinese Management, Xi’an Jiaotong University, Shaanxi, Xi’an, China; Szechenyi Istvan University: Szechenyi Istvan Egyetem, HUNGARY

## Abstract

The maturity and commercialization of emerging digital technologies represented by artificial intelligence, cloud computing, block chain and virtual reality are giving birth to a new and higher economic form, that is, digital economy. Digital economy is different from the traditional industrial economy. It is clean, efficient, green and recyclable. It represents and promotes the future direction of global economic development, especially in the context of the sudden COVID-19 pandemic as a continuing disaster. Therefore, it is essential to establish the comprehensive evaluation model of digital economy development scientifically and reasonably. In this paper, first on the basis of literature analysis, the relevant indicators of digital economy development are collected manually and then screened by the grey dynamic clustering and rough set reduction theory. The evaluation index system of digital economy development is constructed from four dimensions: digital innovation impetus support, digital infrastructure construction support, national economic environment and digital policy guarantee, digital integration and application. Next the subjective weight and objective weight are calculated by the group FAHP method, entropy method and improved CRITIC method, and the combined weight is integrated with the thought of maximum variance. The grey correlation analysis and improved VIKOR model are combined to systematically evaluate the digital economy development level of 31 provinces and cities in China from 2013 to 2019. The results of empirical analysis show that the overall development of China’s digital economy shows a trend of superposition and rise, and the development of digital economy in the four major economic zones is unbalanced. Finally, we put forward targeted opinions on the construction of China’s provincial digital economy.

## 1. Introduction

With the acceleration of industry 4.0, the world has entered the era of digital economy, which is led by the new generation of information and communication technology (ICT). Looking around the world, international relations are strained, the world pattern is changing, economic globalization is facing countercurrents, and the demand market is seriously shrinking. The outbreak of COVID-19 has undoubtedly brought a heavy blow to the global economy, which is already in a downturn. Digital economy emerges with its unique advantages. It is mentioned in the White Paper on The Development of China’s Digital Economy of 2021 released by China Academy of Information and Communication Technology that the total amount of digital economy is up to 39.2 trillion yuan, with an increase of 313.1% compared with 2011 and a ratio of about 39% to GDP. The growth rate of digital economy is much higher than that of GDP. The digital economy has broken down barriers to face-to-face contact, ensured the circulation and smooth flow of domestic and international economy, and played an immeasurable role in the sustained development of major public health emergencies and the post-epidemic era. The digital economy, guided by new development concepts and driven by emerging technologies, relies on the application of information networks and platforms, updates factors and resources, and targets high-quality and sustainable development, as an important cornerstone of building a modern economic system. This creates unprecedented risks and opportunities for stakeholders. The rapid development of digital economy, its connotation continues to expand, deep integration with economic and social industries, promoting a series of digital transformation, and constantly inject new technologies, new models and new industry genes into the transformation and upgrading process of society, industry and enterprise, promoting innovation-driven sustainable economic development model. In addition, while restructuring the economic and industrial structure, these innovations have triggered a new round of technological revolution and generated positive feedback from the technology side, which has a profound impact on enterprises, the public, governments and other stakeholders of various countries.

The core of the digital economy is the continuous innovation and breakthrough of information and communication technology. Digital technology creatively enables economic development not only to demographic dividend, market dividend, wage level, foreign investment and other factors, but also to provide knowledge and data for economic development and industrial upgrading. An important driver of productivity recovery in the United States is the development and application of information and communication technologies [1]. Oliner and Dale W found through calculation that information and communication technology played an obvious role in promoting the economy of the United States [2,3]. Abdul A believed that information and communication technology had a significant effect on the economic growth of India [4]. It can be seen that ICT plays a driving role in the economic growth of both developed and developing countries. On the one hand, the continuous innovation and breakthrough of digital technology itself can give birth to a large number of emerging industries, which indicates that the acceleration of innovation efficiency will promote the emergence of new industries. On the other hand, the integration and iteration of digital technology and traditional technology promote its rapid cross-border integration with various industries, which marks the high permeability of digital technology. These two aspects complement each other and are intertwined, providing the country and society with new technologies, new markets and new labor forces, triggering a series of social changes and technological upgrading, so as to achieve high-quality economic and social development. At the same time, the digital age of massive growth of digital information become the new production factor, subverts the traditional factors of production pattern, elements in great changes of position, to integrate the new elements, accelerate the diffusion of knowledge and technology and achievements transformation, resource allocation optimization, speed up the development of green, clean, intelligent industrial chain, reshape the global industry value chain. The economic growth brought by the digital economy is irreversible. Technological innovation, technological integration and the continuous emergence of information elements make the proportion of output value related to the digital economy in the economy of all countries continue to increase, and further promote the sustainable economic growth.

At present, all countries in the world are actively exploring the development mode of digital economy, hoping to seize the new economic revolution of digital economy and thus lead the trend of world economic development. The development of digital economy brings new changes to the competition pattern among countries and becomes a new driving force for economic development [5]. Therefore, a perfect and scientific evaluation of the development level of digital economy is conducive to accurately exploring the advantages and disadvantages of promoting digital economy, putting forward practical and targeted suggestions for the sustainable and rapid development of digital economy in the future, and building China into a country with strong international competitiveness. It is of irreplaceable guiding and practical significance for China to form a new development pattern and achieve high-quality and sustainable economic development.

This paper will try to use the theory of multi-attribute decision to calculate the development level of China’s digital economy and on this basis, put forward some specific suggestions for development. The rest of this paper is organized as follows: The literature review part sorts out the latest research achievements of relevant scholars in the field of digital economy. In the part of model design of comprehensive evaluation index system, this paper constructs an index screening model and comprehensive evaluation model, and expounds the specific steps in detail. In the part of empirical analysis, this paper will conduct empirical evaluation on the development level of digital economy according to the method of model construction, and analyze and classify the evaluation results. The conclusion part summarizes the research results of this paper and puts forward relevant suggestions.

## 2. Literature review

### 2.1 Definition of digital economy

As a key measure to lead the development of the global economy, the digital economy has an important impact on many aspects such as the improvement of international competitiveness, the transformation of the manufacturing industry, and the upgrading of the industrial structure. Digital economy was coined as a technical term around 1990. In 1996, Don Tapscott called digital economy the era of networked intelligence, believing that digital economy is a network system constructed by human beings through technology, which links knowledge, skills and innovation to promote wealth and social development of creative breakthroughs [6]. Moulton believe that information technology and e-commerce are digital economy [7]. Brynjolfsson and Kahin proposed that digital infrastructure is digital economy [8]. With the outbreak of the scientific and technological revolution, the connotation of digital economy is gradually expanding, and commodity trading and services based on information and communication technology should also be included [9]. As a new element, data creates infinite possibilities for products and services [10], and subversively changes the way of creating economic benefits [11]. New products and services are the reason why the digital economy is called the "new economy" [12]. Bukht and Heeks defined digital economy as the economic activities caused by digital technology and the products or services applying digital technology, and its core is the IT or ICT department that produces basic digital goods and services [13]. Some scholars believe that scientific and technological innovation is the core of the development of digital economy [14]. The specific form is reflected in the realization of Commodity Exchange by digital means [15], which has become the representative feature of the emergence of new economic forms [16]. The UK government proposed that digital economy not only includes digital goods and services, but also digital enterprises that use digital technologies for transformation and upgrading [17]. Although there is no unanimously recognized concept of the digital economy, the academic community generally recognizes the definition proposed by the G20. It believes that the digital economy is a series of economic activities that use digital knowledge and information as key production factors and use information and communication technology to promote the optimization of economic structure and improve efficiency.

### 2.2 Evaluation of the digital economy

At present, there are mainly authoritative institutions and researchers on measuring digital economy, which can be divided into the following three categories: the first category mainly includes the United Nations International Telecommunication Union, the Organization for Economic Cooperation and Development(OECD), the US Department of Commerce, the World Economic Forum and so on. OECD, the first international organization to study digital economy, is the most representative and authoritative [18]. Its research on digital economy is long-term and forward-looking. OECD’s definition of digital economy connotation is based on broad perspective, including inclusive perspective, accounting perspective and preliminary measurement perspective [19]. The second category is research reports on the development of the digital economy issued by domestic institutions and companies in China. There are mainly Shanghai Academy of Social Sciences, China Academy of Information and Communication (CAICT), Xinhua Three Group, Tencent and so on. Among them, the Digital Economy Index(DEI) published by CAICT is the most widely spread and most reliable. The third category is the academic researchers. From a theoretical perspective, John Haltiwanger and Ron believed that digital economy should be measured from five aspects: IT infrastructure, e-commerce, industrial structure and labor characteristics, but did not measure [20]. Wan Xiaoyu, Luo Yanqing and Yuan Ye made a theoretical analysis of the relevant connotation of digital economy from three aspects of digital input, digital output and digital environment [21]. The operability of empirical analysis was almost impossible for the index system constructed by them because it ignored the availability of data and the actual situation of each province. But it provides useful reference for the study of digital economy evaluation. From the practical point of view, the measurement methods of digital economy mainly include scale measurement and quantification by constructing index system, which is a beneficial preliminary exploration for the measurement research of digital economy in the later period. Vincent Didiek made a preliminary quantitative analysis of digital economy [22]. Sarma proposed to use Euclidean distance joint efficacy function to solve the measurement problem of digital economy [23]. HB Zaman adopted structural equation model to measure digital economy [24]. Juha Itkonen believes that the premise of accurately measuring the digital economy is to create a perfect statistical system [25]. Andriy Stavytskyy used panel data to empirically analyze the digital economy and society Index (DESI) and found that the index was positively correlated with the consumption index and negatively correlated with the unemployment rate [26]. Sidorov Anatoly established a comprehensive index model of regional digital economy development level by using functional network [27]. Zhang Wei, Zhao Siqi, Wan Xiaoyu and Yao Yuan measured the digital economic development index of 30 cities in China from the three dimensions of digital infrastructure, digital industry, and digital integration, uses panel data of 30 cities in China from 2015 to 2019 to construct an econometric model for empirical analysis, and verifies the mediating effect of technological progress between the digital economy and high-quality economic development [28]. In addition, Mueller measured the digital economy from the enterprise level and substituted the development of digital economy in different regions by discriminating the market value of enterprises involved in digital technology [29]. Although some achievements have been made on the measurement of the digital economy, scholars only select the basic indicators they need when establishing the measurement system of the digital economy, and rarely measure the development level of the digital economy comprehensively.

### 2.3 Related impacts of the digital economy

Most research focuses on the impact of digital technology, information technology and the Internet on economic growth and enterprise. On the one hand, the gradual upgrading of ICT and digital talents has reshaped the original pattern of manufacturing industry and promoted it to develop in a more unified and united direction [30], thus promoting the regional economic growth [4,28,31–33] and productivity improvement [34,35]. Aris Pagoropoulos proposed that digital technology can accelerate the emergence of services and products in the tertiary industry and produce inestimable economic benefits [36]. Oliner and Sichel verified its contribution to economic growth by reclassifying the input of computer-related industries [2]. Digital economy has also shown significant effects in reducing market friction [37] optimizing transport and logistics complex [38], and promoting the formation of new human capital [39]. On the other hand, the application scenarios of digital technology broaden and redistribute the market share of enterprises [40], and enterprises that use more information technology will rely more on this technology to improve their own production efficiency and organizational efficiency [40,41]. Information technology can improve the ability of enterprises to actively innovate [42,43], but Paunova and Rollob found that this driving effect has firm heterogeneity [44]. Autor believe that the development of digital technology promotes some leading enterprises in the industry to obtain large profits, causing the gap between enterprises to gradually increase [45]. Some studies also believe that the main impact of digital economy on enterprises is to significantly reduce various costs of enterprises [46]. In addition, some scholars study digital economy from a special perspective. Vasilescu, Serban and Dimian used statistical analysis to clarify EU citizens’ perceptions on digitization. Through cluster analysis and logistic regression, the influence of relevant factors on citizens’ digital cognition and the influence of digitally disadvantaged groups and digitally disadvantaged countries on the exposure of digital divide were respectively quantified [47]. The results show that citizens’ awareness of digitisation will have a profound impact on the EU’s economy and society in the next period [47]. This study is a useful exploration for the future development of the European Union and also has certain reference significance for other countries.

### 2.4 Multiple attribute decision theory

The development of multi-criteria decision making originated from the optimal concept proposed by Pareto in the late 19th century, and it was the multi-criteria decision making conference held in Columbia University in the 1970S that really attracted attention [48,49]. The evaluation of digital economy includes a limited number of regions, so it can be regarded as a multi-attribute decision problem. The core process of solving multi-attribute decision making problem is index system construction method, attribute weight assignment and comprehensive evaluation. These three key steps are discussed below.

(1) The method of weighting is divided into single weighting method and comprehensive weighting method. Due to the limitation of single weighting method, integrated weighting method is commonly used in existing papers. Combined weighting solves the shortcomings of subjective and objective weighting, synthesizes the advantages of both, not only combines the experience and actual situation of subjective weighting decision-makers, but also considers the relationship between the actual data of objective weighting. Sun Bing and Su Xiao used maximum deviation method to combine objective weight and subjective weight to determine the optimal combination weight in shipyard supplier selection [50]. Wang and Lee combined the subjective preference of the judge with the fuzzy entropy of the discriminant matrix to calculate the index weight [51]. Hua Zhao and Ze Shui Xu proposed a method based on minimum bias to integrate subjective preference and objective preference and obtain portfolio weight [52]. Wang et al. used FAHP method and fuzzy preference to construct a combined weighting model to calculate attribute weights [53]. In some studies, the method of integrating subjective and objective weights was proposed to calculate the weight of combinations [54]. Wang and Luo proposed an interactive weighting method that comprehensively considers correlation coefficients and standard deviations between attributes [55]. Chao and Yang S established an attribute weight feedback model based on group consensus, which improved the accuracy of group opinion [56]. Duan Chuanqing improved the traditional entropy method by using the correlation coefficient, and then proposed a combined weighting method combining the preference values of decision makers, and verified the effectiveness of this method [57].
(2) In the process of solving multi-attribute decision making problems, it is an important prerequisite for obtaining correct decision making results that the index system constructed has scientificity and comprehensiveness. The existence of multi-attribute features in a problem will inevitably lead to information overlap and interference among attributes. However, traditional index screening methods including analytic hierarchy process (AHP), expert investigation, regression analysis, fuzzy evaluation, etc., cannot deeply reveal the internal relationship between attributes and the realization of goals, let alone improve the goals according to the results of regular analysis [58,59]. Scholars find that rough set theory has incomparable advantages in this kind of problems. In the 1980s, Professor Z Pawlak of Warsaw University of Technology first proposed the rough set theory, which, as a mathematical tool, is specially used for the analysis of imprecise problems. In this method, the collected samples are formed into a sample set, and an initial knowledge system is constructed. The redundant attributes of the system are eliminated by attribute reduction, so the optimal knowledge system including key attributes is obtained, and the connections and potential laws between data are effectively mined. Rough set theory has been widely used in management ability evaluation, coal mine production efficiency, highway site planning, economic evaluation, urban comprehensive strength evaluation and other related fields to solve multi-attribute decision making problems because it does not need to receive prior knowledge and all kinds of conditions.

It can be divided into two categories based on index selection and system construction method. The first is to use a single screening method. Jia Yajuan, Ning Zekui and Yang TianRong constructed the competency evaluation index system of new professional farmers by using analytic hierarchy Process (AHP) on the basis of Delphi expert investigation [60]. Chi Guotai and Chen Honghai proposed an index screening and weighting model based on information sensitivity, which avoided the problems of failure to assign weights and reasonable screening indexes existing in the existing principal component analysis [61]. Shen Zhenyao and Yang Zhifeng proposed to use grey correlation analysis to delete weak correlation indicators and screen indicators [62]. Wu Huawen adopted the factor analysis method to establish a railway industry evaluation index system including four aspects, and elaborated the measurement methods of relevant indicators [63]. Li Xiaohan, Hu Qizhou and Zhou Hao took passengers, enterprises and government as tripartite subjects and established an index system to measure railway passenger service quality by using rough and intensive simplification theory [64]. The second is to combine two or more kinds of mathematical models to establish evaluation system, and realize the complementarity of advantages between different analysis methods. Wang Hongzhi, Gao Xuedong and Lai Yuanyuan combined rough set theory with grey theory and proposed an improved reduction algorithm using the fusion attribute of grey rough set, and demonstrated the timeliness of the algorithm through numerical examples [65]. Yang Haixia, Li Chenyu et al. proposed the FP-tree algorithm in order to solve the problem of strong correlation among indicators in complex multi-attribute evaluation problems [66]. Zhang Zhaoyang, Zhao Tao and Wang Chunhong proposed a method of index reduction based on rough set theory [67]. Gao Jie, Sun Linyan and Li Manyuan proposed that in the AHP method, interval estimation was used to eliminate indicators with weak weights [68]. Zhou Ying, Wang Hongzhi and Chi Guotai used the quantitative method of cluster analysis combined with factor analysis to screen indicators in the process of constructing green industry evaluation system [69].

(3) The continuous deepening of comprehensive evaluation methods benefits from the continuous enrichment and maturity of statistics and decision theory. Pareto expands the range of decision-making objectives from single to multiple. At the same time, Pareto optimality theory gave birth to multi-objective decision making theory using comprehensive evaluation [70]. In 1992, Dyer prospected and discussed the development of multi-attribute decision theory in the next decade [71]. Wallenius analyzed the development trend of multi-criteria decision making on the basis of sorting out existing researches [72]. Johansson, Falkman, Wang et al used bayesian network method to comprehensively evaluate the threat degree of air defense targets [73]. Some scholars use technique for order preference by similarity to an ideal Solution (TOPSIS) to comprehensively evaluate the degree of target threat. In the decision-making process of actual situations, fuzzy information often appears. Jain proposed a fuzzy number sorting method based on the idea of fuzzy maximization, and verified the accuracy of this method in decision-making problems [74]. In 1985, Based on the analysis of five fuzzy sorting methods, Chen proposed the idea of maximization and minimization to sort fuzzy numbers, and applied this method to triangular and trapezoidal fuzzy numbers [75]. Wang and Yang used Euclidean distance to construct the centroid fuzzy number formula and sort the fuzzy numbers, improving the accuracy of the sorting method [76]. Ml et al. believed that fuzzy comprehensive evaluation method had obvious advantages in theory and application, and applied it to the study of real estate investment risk [77]. Chang applied triangle fuzzy number to reflect process uncertainty, combined fuzzy theory with evaluation method, and proposed an improved VIKOR method to evaluate service quality [78]. Wen Haili and Xia Weiyi analyzed the correlation between problems existing in characteristic towns in Guangxi and used VIKOR method to evaluate the construction of 14 characteristic towns in Guangxi [79]. Pan Yahong and Geng Xiuli improved the traditional VIKOR method and proposed a modular random multi-criteria compromise solution ranking method to avoid information loss of mixed evaluation information in the assembly process [80].

### 2.5 Literature summary

To sum up, researches on digital economy have made a lot of progress, but most of them are limited to theoretical researches, and few are explored from the empirical perspective. Moreover, the index selection of digital economy index system lacks comprehensiveness and covers a narrow range. On the one hand, for the construction of digital economy evaluation index system of selection index and index system of the design of the different scholars differentiation phenomenon is serious, selection criteria, and the lack of a wide range of expert advice, horizontal contrast, unified index system has been widely accepted by the academia has not formed, the evaluation results credibility is not high. On the other hand, as for the comprehensive evaluation method, it is generally used to calculate the index by linear weighted sum of weights and data, and no scientific and reasonable comprehensive evaluation model has been established. In the context of China’s internal circular mechanism of accelerating the cultivation of a complete domestic demand system, the digital economy, represented by a new generation of emerging technologies such as big data, mobile internet of things and artificial intelligence, drives digital transformation. To study how to establish and measure the index system of digital economy is a major task to be urgently solved at present, and also a major mission entrusted by the new era.

**Fig 1:**
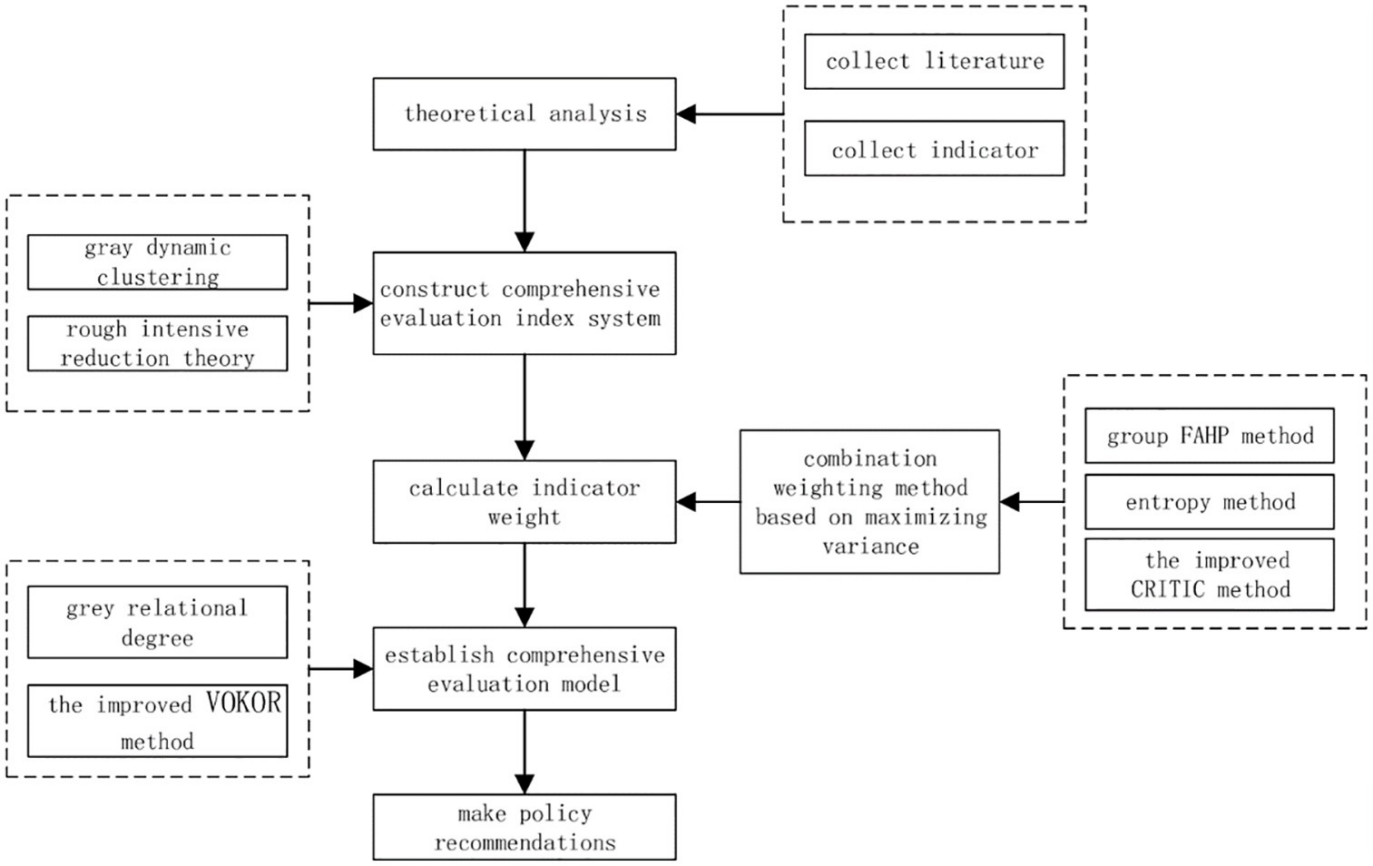
Full text implementation flowchart.

In view of this, this paper takes the development of digital economy in 31 Provinces of China from 2013 to 2019 as the empirical research object. Firstly, based on the relevant results of previous studies and combined with the characteristics of the development of digital economy, the indicators with significant representativeness were selected to construct the index system of this paper. Secondly, in order to reduce information duplication among indicators and build a reasonable and objective digital economy indicator system, the indicators are preliminarily screened by combining grey clustering [81] and rough intensive reduction theory. Thirdly, a combination weighting method based on variance maximization is used to determine the weight of indicators in order to comprehensively consider the experts’ experience knowledge and the potential information of mining data. Finally, the improved VIKOR method is used to comprehensively evaluate the development level of digital economy. The specific flow chart is shown in Fig 1.

## 3. Model design of digital economy comprehensive evaluation index system construction

### 3.1 Index screening method based on grey clustering and rough set reduction theory

**Fig 2:**
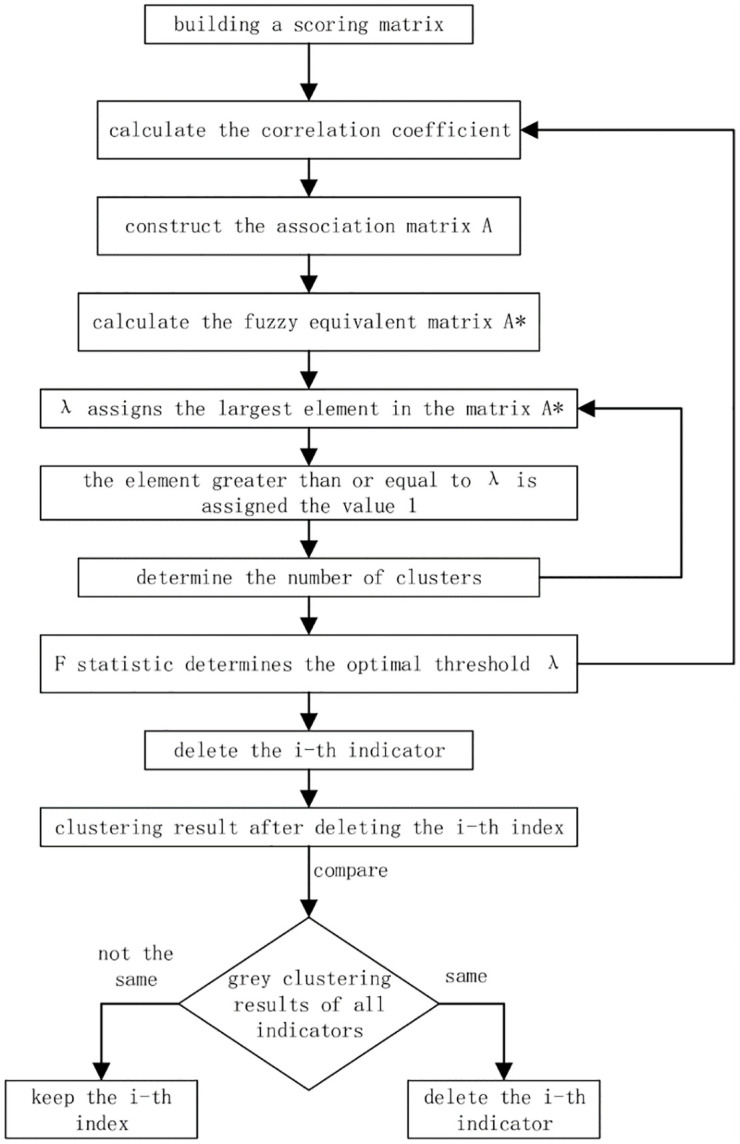
Indicator screening flow chart.

Because grey correlation analysis has low requirements on sample data, it is not limited to the number and distribution of data [82]. The rough intensive reduction theory [83] is a natural data mining method that can easily process data without any prior knowledge. Therefore, in order to optimize the index system of digital economy, this paper adopts the method of combining the grey dynamic clustering and the rough set attribute reduction theory to screen indicators. The detailed process is shown in Fig 2.

#### 3.1.1 Collection and preliminary screening of indicators

(1) Collection indicators

On the basis of reading a large number of relevant literatures, this study adopted extensive reading, intensive reading, browsing and other methods to integrate and screen the literatures involved, and finally selected a total of 50 literatures closely related to digital economy evaluation. Firstly, the indicators in the selected literature were collected manually and the frequency of occurrence of each indicator was calculated. Select 33 indicators whose frequency is greater than or equal to 4 times. Then, due to the low frequency of some important indicators, the indicators that appeared less frequently in the first screening and were not selected were added. Finally, 38 indicators are selected and combined with the characteristics of the digital economy to construct the evaluation index system of the digital economy, as shown in Table 1.

**Table 1:** The comprehensive evaluation index system of digital economy development level.

First-Level Index	Second-Level Index	Third-Level Index	Index Screening Results
Digital Innovation Capability (A)	digital talent investment (*A*_1_)	per capita investment in education (*A*_11_)	retain
		operating expenses of education (*A*_12_)	retain
		the proportion of college degree or above in total employment (*A*_13_)	retain
	digital technology investment (*A*_2_)	scientific career fee (*A*_21_)	retain
		technology market turnover (*A*_22_)	retain
		the proportion of scientific and technological employees (*A*_23_)	retain
		the number of patents granted (*A*_24_)	retain
	capital investment (*A*_3_)	investment intensity of R&D expenditure (*A*_31_)	retain
	data input (*A*_4_)	data factor input intensity (*A*_41_)	retain
		data element input density (*A*_42_)	retain
Digital Infrastructure (B)	mobile base investment (*B*_1_)	mobile telephone exchange capacity (*B*_11_)	retain
		mobile phone penetration rate (*B*_12_)	retain
		the number of fixed phone users (*B*_13_)	retain
	internet infrastructure investment (*B*_2_)	internet broadband access port (*B*_21_)	retain
		length of optical cable (*B*_22_)	retain
		the number of domain names owned (*B*_23_)	retain
		internet penetration rate (*B*_24_)	retain
		the number of websites owned (*B*_25_)	retain
		ratio of IPV4 addresses (*B*_26_)	retain
		mobile broadband penetration rate (*B*_27_)	delete
National Economic Environment and Digital Policy Guarantee(C)	economic environment (*C*_1_)	GDP per capita (*C*_11_)	retain
	digital economy development environment (*C*_2_)	digital economy industrial output value occupies GDP proportion (C_21_)	retain
		digital consumption as a share of GDP (*C*_22_)	retain
	digital policy guarantee (*C*_3_)	the proportion of internal EXPENDITURE of R&D expenditure to GDP (*C*_31_)	retain
		the proportion of fixed asset investment in digital economy in GDP (*C*_32_)	retain
		the proportion of public security expenditure in general public budget expenditure (*C*_33_)	retain
Digital Fusion Application(D)	personal digital fusion application (*D*_1_)	the number of computers owned (*D*_11_)	retain
		the actual number of digital TV users (*D*_12_)	retain
	enterprise digital integration application (*D*_2_)	proportion of enterprises with e-commerce transaction activities (*D*_21_)	retain
		the number of informationized enterprises (*D*_22_)	retain
		the number of computers used by enterprises (*D*_23_)	retain
		the number of web sites owned by enterprises (*D*_24_)	retain
		software business revenue (*D*_25_)	retain
	digital transaction (*D*_3_)	the proportion of e-commerce sales in regional GDP (*D*_31_)	retain
		express quantity (*D*_32_)	retain
		online retail sales as a percentage of GDP (*D*_33_)	delete
		digital Financial Inclusion Index (*D*_34_)	retain
		the proportion of e-commerce purchase in regional GDP (*D*_35_)	retain

(2) Preliminary construction of the index system

**Fig 3:**
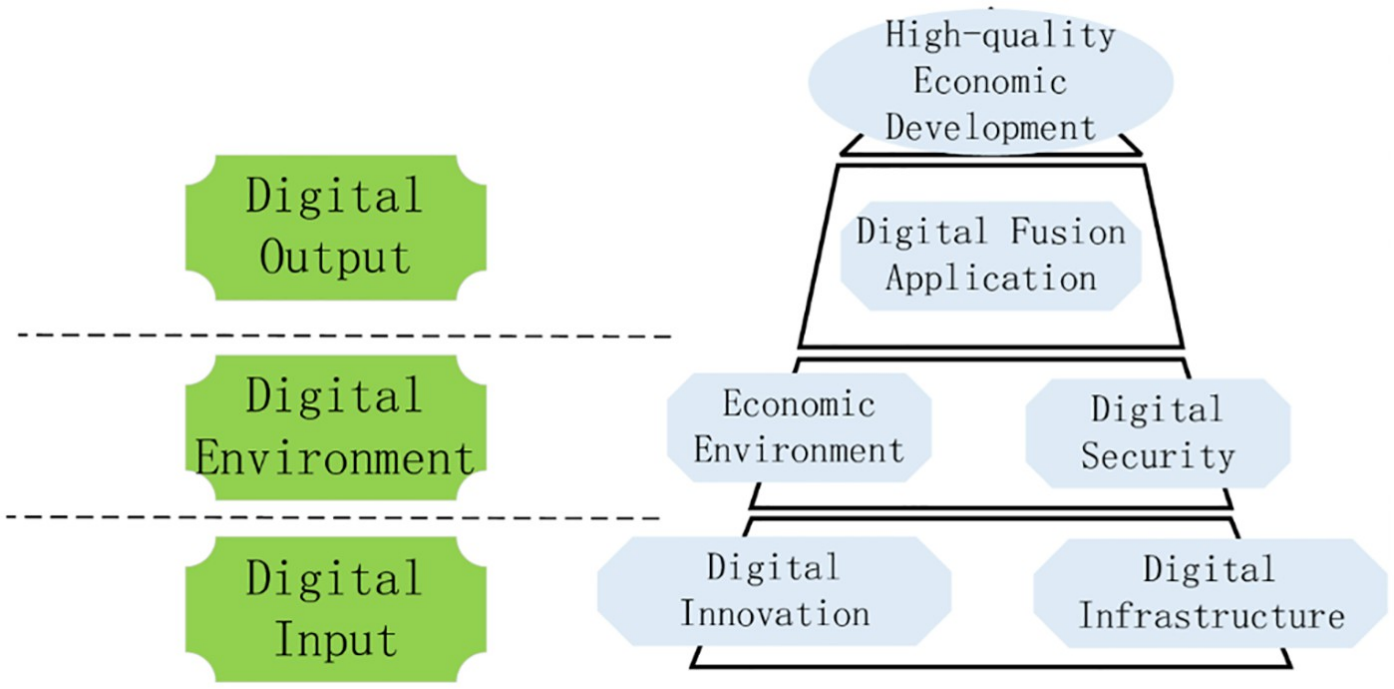
Logical framework of digital economy index system.

The establishment of an evaluation system from a diversified and systematic perspective can not only reflect the regional differences and characteristics of the development of digital economy, but also make the development level of digital economy comparable, thus guiding the future direction of the development of digital economy. Based on the analysis and comparison of the existing literature, the paper aims to clarify the specific content of digital economy, reflect the essence of digital economy and reflect the development of digital economy comprehensively. From the perspective of input-output, this paper establishes the index system to measure the development of digital economy, based on the development law and connotation of digital economy. The digitalization policy guarantee capability is included in the index system, and the vertical development of digital industry is promoted by combining the inherent meaning of digital quality and quantity. Then reveal the digital transaction and its influence in the process of digital input to digital output. Reflect the value of digital technology, show the internal logic and way of digital empowerment, and realize the high quality economic development during the "14th Five-year Plan". The logical framework of the digital economy indicator system is shown in Fig 3.

1) Digital input

Digital input directly reflects the hardware and software conditions and resource endowment conditions of digital economy in each region. On the one hand, digital innovation is a combination of old and new production factors that drive the development of digital economy. It represents the power source to maintain the sustainable development of digital economy. Maintaining high innovation ability is the key aspect to stimulate the potential development of digital economy in the future. General Secretary Xi Jinping has stressed that development is the top priority and talent is the primary resource. We need to unleash the huge potential of science and technology as the primary productive force, accelerate the formation of a digital economy led by innovation, and create a contingent of big data talents. It can be seen that talent, technology and data are important ways to realize regional digital economic growth. In addition, the intensity of capital investment in each region also directly affects the development degree and speed of digital economy, an emerging economy. Therefore, digital innovation investment mainly includes digital data investment, talent investment, digital technology investment and capital investment, which provides an inexhaustible power to stimulate the development vitality of digital economy and support the digital economy to an unprecedented "peak". On the other hand, digital infrastructure construction mainly refers to the hardware and software equipment providing basic services for information and communication activities, which is the pillar of new infrastructure construction and the prerequisite of digital activities. The core of the development of digital economy is the Internet. The intelligent world of the Internet of everything requires portability, convenience and small size. The continuous upgrading of mobile terminal equipment and network technology promotes the process of digitalization. In this paper, digital infrastructure construction is represented by mobile infrastructure investment and Internet infrastructure investment.

2) Digital environment

Digital environment is a necessary condition and strong support for the formation of digital economy. The construction of digital economic environment is based on the establishment of a thriving digital economic development environment for digital policy protection, and constantly improve the level of digital application. Economic booming in the region is bound to be mature and efficient digital policy in order to create superior digital economy development environment, and the lagging economic development provinces for policy making and lack of experience in the digital economy, limited by its economic environment, the development of the digital economy has lagged, no doubt. Policy system is an important ballast stone to ensure the digital economy environment and an environmental guarantee to promote the stable development of the digital economy. Digital environment includes economic environment, digital economy development environment and digital policy protection environment.

3) Digital output

Digital input provides the conditions for the development of digitalization, digital environment creates a space atmosphere for the development of digital economy, and digital output should be displayed in the application of digitalization. Digital output is reflected in the process of digital upgrade when enterprises provide digital services, and the whole society uses digital technology or digital platform to carry out digital transactions. Digital service is the concrete embodiment of the transaction process of digital economy, that is, it embodies the value of digital service. The performance of digital economy is not obvious at the initial stage of development. Only when it has developed to a certain stage will it show its advantages and release the potential of promoting economic growth. Digital services, digital products and services including product permeability of consumption level, the scale of the digital economy + services such as body content, it merge with related industries to promote digital economic growth brings infinite opportunities, such as retail, catering, education and other digital development of traditional industries, platform economy, share with their new consumption form to meet the diverse needs of the public, Even private "tailor-made". This paper mainly measures the intensity of enterprises’ investment in digital transactions under the urging of digital investment and environment. Therefore, the digital output will be measured from three aspects: the basis of personal digital transaction, the basis of enterprise digital transaction and the impact of digital transaction.

(3) Preliminary screening

Among the 38 indicators selected in the previous step, some important indicators cannot obtain data and can be replaced by similar indicators; the index with missing value can be complemented by interpolation method; for indicators with complete data, you can perform the next step of filtering.

#### 3.1.2 Gray dynamic clustering

(1) Establish the scoring matrix

Six senior experts in the field of digital economy were invited to score the constructed evaluation index system from four aspects of "typicality, scientificity, systematicness and sustainable development". Each item is 25 points out of 100 points. In this paper, the index system of digital economy is set as a multi-attribute decision information system *S* = {*U*, *C*, *V*, *P*}. Where *U* is the whole sample set, indicating that m experts are scoring. *C* is a conditional attribute set that reflects the characteristics of an object, indicating that there are n indicators in the index system. *C* = *C* ∪ *D*, *C* and *D* are conditional attribute sets and decision attribute sets respectively. *V* is the set of attributes. *P* is an information function, representing the score value of the i-th expert for *j* indicators.

(2) Data standardization

Because the indicators involved in this paper are all positive indicators, only the standardization of positive indicators is introduced.

Suppose *y*_*ij*_ is the original scoring value of the j-th digital economic index by the i-th evaluation expert, and the standardized scoring matrix is *Y* = [*y*_*ij*_], where *i* = 1, 2, …, *m*, *j* = 1, 2, …, *n*. *M* represents the number of scoring experts, and *N* represents the number of indicators in the index system. The standardized formula is:

yij=yij-min1≤i≤myijmax1≤i≤myij-min1≤i≤myij (1)

(3) Establish grey correlation matrix

Suppose *Y* = {*y*_1_(1), *y*_2_(2), ⋯ *y*_*m*_(*n*)} represents the scoring sequence, *α*_*ik*_(*j*) represents the correlation coefficient between *y*_*i*_ and *y*_*k*_ in the evaluation of the j-th index (When *i* = *k*, *α*_*ik*_ = 1), and the resolution ρ = 0.5. Formula (2) calculates the grey correlation coefficient, and Formula (3) is the grey correlation matrix *C*.


αikj=miniminjykj-yjj+0.5*maximaxjykj-yjjykj-yjj+0.5*maximaxjykj-yjj (2)

C=α11α12⋯α1mα22⋯α2m⋱⋮αmm (3)

(4) Determine the optimal threshold *λ*

In this paper, F statistics are used to calculate the optimal number of categories. The formula of F statistic is:

F=∑i=1rniyi--y-2/r-1∑i=1r∑j=1niyji-yi-2/n-r (4)

In the above formula, *λ* is the threshold and λ = [0, 1]. The value of *λ* can be obtained from the specific calculation process, and the classification of different threshold *λ* is different. *r* represents the number of categories at the threshold *λ*. *n*_*i*_ stands for the number of evaluation objects in class *i*(*i* = 1, 2, ⋯, *r*). ${\left\|\overset{-}{y^{i}}\right.-\left.\overset{-}{y}\right\|}^{2}=\left\|\overset{-}{y^{i}}\right.-\left.\overset{-}{y}\right\|=({\sum_{h=1}^{n}\left(\bar{y_{h}^{i}}-\bar{y}\right))}^{1/2}$ stands for the Euclidean distance between the cluster center in class *i* and the total sample cluster center, *$\left\|y_{j}^{i}\right.-\left.\overset{-}{y^{i}}\right\|=({\sum_{h=1}^{n}\left(y_{jh}^{i}-\bar{y_{h}^{i}}\right))}^{1/2}$* indicates the Euclidean distance between the normalized data and the clustering center of the class it is in. The larger the value of *F* is, the larger the interval between different categories is. At this time, the classification is the optimal classification, and the corresponding *λ* is the optimal threshold.

#### 3.1.3 Rough set index reduction theory

The rough intensive reduction theory is to judge whether each indicator or attribute needs to be reduced on the basis of the unchanged number of original sample classification, remove redundant information and retain the integrity of the original information [84].

(1) Assuming that an information system is *S* = {*U*, *C* = *A* ∪ *D*, *V*, *F*}. This expression contains the following sections:
*S*: represents an information system;
*U*: *U* = {*x*_1_, *x*_2_ *⋯*, *x*_*k*_} is the domain of theory, representing the collection of all objects.
*C*: *C* = {*c*_1_, *c*_2_ *⋯*, *c*_k_} is a set of attributes of an object. *C* = *A* ∪ *D*, *A* ∩ *D* ≠ *∅*, *A* and *D* respectively represent conditional attributes and decision attributes.
*V*: *V*_*A*_ = {*V*_*a*_|*a* ∈ *A*} is the set of condition attribute values, *V*_*D*_ = {*V*_*d*_ |*d* ∈ *D*} is the set of decision attribute value.
*F*: *F* is a function that determines attribute values in the way of mapping, which can be expressed as *F*: *U* × *C* → *V*.
(2) If there is a decision information system in (1) above. *∀a* ∈ *A*, *∀z*, *w* ∈ *U*, *F*(*z*, *a*) ∈ *V*_*A*,_ *∀O* ⊆ *A*, *O* ≠ *∅*, then the indiscernible relation IND defined in U can be expressed as:
$$IND\left(O\right)=\{(z,w)\in U\times U|F\left(z,l\right)=F\left(a,l\right),\forall l\in O\}$$

*IND*(*P*) represents a indiscernible relationship about *P*(*∀P* ⊆ *Q*), which is a division of the domain *U*, and can be abbreviated as *U*/*IND*(*P*) = {*Y*_1_, *Y*_2_ ⋯, *Y*_*k*_}. Suppose a set of equivalence relations defined on *U* is *D*(*d* ∈ *D*). If the equation *ND*(*D*) = *IND*(*D* − {d}) does not exist, then *d* is said to be necessary *D*, otherwise it is redundant. *D* is independent if any *d* is not unnecessary in *D*. If two equivalent relations *A* and *B*(*A* ∈ *B*) are defined on *U*, *A* is independent and *IND*(*A*) = *IND*(*B*) is true, then *A* is called the reduction of the field *U* on the attribute set *B*.

### 3.2 Combination weighting method based on variance maximization

In order to integrate the advantages of subjective and objective weighting, some scholars proposed the use of interactive weighting [85], combined weighting method [86,87], combined weighting TOPSIS method [88–90], multiplicative integrated weighting method [91], etc. Although the above methods solve the shortcomings of subjective and objective weight assignment methods to a certain extent, the selection of subjective and objective weight determination methods is arbitrary, and there is no appropriate method for the integrated allocation of the two weights. Based on the demonstration of relevant literature [92,93], this paper will use the method of maximum variance to synthesize the subjective and objective weight.

#### 3.2.1 Subjective weighting method based on group FAHP

The analytic hierarchy process has some problems, such as it is difficult to realize the consistency test of judgment matrix and there is no scientific basis, it cannot find the elements causing the inconsistency of judgment matrix, and the consistency of human thought is quite different from the consistency of judgment matrix [94]. Therefore, researchers proposed fuzzy analytic hierarchy Process. The steps of group fuzzy analytic hierarchy process are as follows:

(1) Establish an index system

This content is described in detail in section 3.2.1 and will not be repeated here.

(2) Construct fuzzy complementary judgment matrix

The fuzzy judgement complementarity matrix represents the comparison of the importance degree between the indicators in the next level for the indicators in the previous level. Suppose that an index at the upper level is *A*, and the index at the lower level is *B*1, *B*2, ⋯ *Bn*, then the relative importance of the indexes is:

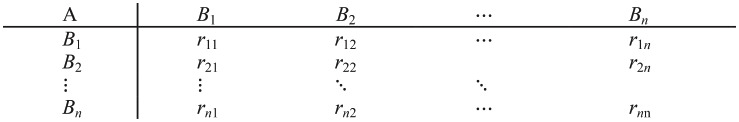


The element *r*_*ij*_ in the matrix represents that when the importance of *B*_*i*_ and *B*_*j*_ is compared with the element *A* at the upper layer, elements *B*_*i*_ and *B*_*j*_ have one degree of membership that is more important than the other. This paper uses the (0.1–0.9) scale to measure the importance between the two elements, as shown in Table 2.

**Table 2:** (0.1–0.9) scale method meaning.

scale	Meaning
0.1	*j* is more strongly important than *i*
0.2	*j* is more important than *i*
0.3	*j* is obviously more important than *i*
0.4	*j* is slightly more important than *i*
0.5	*i* is as important as *j*
0.6	*i* is slightly more important than *j*
0.7	*i* is obviously more important than *j*
0.8	*i* is more important than *j*
0.9	*i* is more strongly important than *j*
the reverse comparison	If the judgment value of the comparison between factor *i* and *j* is *r*_*ij*_, then the judgment value of the comparison between factor *j* and *i* is *r*_*ji*_ = 1 − *r*_ij_

(3) Consistency test and adjustment of fuzzy complementary judgment matrix *R*
  1) Assuming that *R* is the n-order fuzzy complementary matrix, the initial fuzzy judgment matrix is specified as *R*, and the critical value of the consistency index is determined as *Y*(*Y* > 0, *Y* = 0.1).
  2) Assuming that the weight vector of R is *W* = (*w*_1_, *w*_2_, ⋯, *w*_*n*_)^*T*^, the weight vector *W* of fuzzy complementary judgment matrix *R* can be calculated as follows:
wi=1n-12a+1na∑k=1nrik,(a≥n-12) (5)
    The smaller the value of *a* is, the more attention decision-makers pay to the importance difference between factors.
  3) Assuming that the indicator to measure the degree of consistency is *d*. if *d* < Y, go to step 7. otherwise, go to Step 4. The calculation formula of consistency indicator *d* is:
d=2nn-1n-2∑i=1n-1∑j=i+1n∑k=1k≠i,jnrij-rik+rkj-0.5 (6)
  4) Assumption for judging matrix *R*, the characteristic matrix for R*, deviation matrix for *E*, calculate |*d*_*st*_|.
$$E={\left(d_{ij}\right)}_{n\times n}=R-R^{*}$$ (7)
dst=maxdij:i<j,j∈N (8)
  In Eq (7), the matrix E is the antisymmetric matrix. $R^{*}={\left(r_{ij}^{*}\right)}_{n\times n}={\left(a\left(w_{i}-w_{j}\right)+0.5\right)}_{n\times n}$.
  5) Take the first several elements from the largest absolute value of *d*_*st*_ during each adjustment. If *d*_*st*_<0, *r*_*st*_ is increased and *r*_*ts*_ is reduced. If *d*_*st*_>0, *r*_*st*_ is reduced and *r*_*ts*_ is increased. When adjusting, assume that the increase or decrease is *θ*, its value should be as appropriate as possible, and the value should be controlled within the range of [0, 1]. At this point, the adjusted fuzzy complementary judgment matrix $R^{\prime }={\left(r_{ij}^{\prime }\right)}_{n\times n}$ is obtained.
  6) Repeat steps in 2) and 3).
  7) After the adjustment, a satisfactory and consistent fuzzy complementary judgment matrix *S* is obtained.

(4) Determining expert weight
  1) Expert subjective weight. The suggestions and scores given by experts are influenced by their own influence and the decision results given, that is, there are subjective and objective levels. In this paper, three senior experts in digital economy research are invited to give a score. The set of experts is represented as *P* = {*P*_31_, *P*_32_, *P*_3_}. Since the academic ability and knowledge reserve of each expert are almost the same, the subjective weight of all experts is determined to be the same, and the fuzzy judgment matrix is expressed as:
$$P=\left[\begin{array}{ccc} 1/2 & 1/2 & 1/2\\ 1/2 & 1/2 & 1/2\\ 1/2 & 1/2 & 1/2 \end{array}\right]$$
  After calculation, the subjective weight vector of each expert can be obtained as ϑ = (0.333, 0.333, 0.333).
  2) Objective weight of experts. Three judgment matrices $R_{A}^{1}$, $R_{A}^{2}$ and $R_{A}^{3}$ are obtained from the initial fuzzy matrix scored by the experts through step (3). The consistency indexes $d_{A}^{1}$, $d_{A}^{2}$ and $d_{A}^{3}$ of the three matrices can be calculated. Formula (9) is used to calculate the objective weight vector *σ*_*k*_ of experts.
σk=(dAk)-1∑k=13(dAk)-1,k=1,2,3 (9)
  3) Expert weight *M*_*k*_. The expert weight can be calculated from Eq (10).
Mk=αϑk+1-ασk,0≤α≤1 (10)

In the above formula, *α* represents the importance of expert subjective weight. Selection of *α* = 1/2.

(5) Determine the weight of indicators

The three judgment matrices $R_{A}^{1}$, $R_{A}^{2}$ and $R_{A}^{3}$ obtained in step (3) can be transformed into the comprehensive judgment matrix $R_{A}^{\prime }$ through Eq (11). $r_{ij}^{\prime }$ represents the element in the synthetic judgment matrix $R_{A}^{\prime }$. Formula (5) is used to calculate the weight of the index *Q*_*i*_.


rij′=∑k=13τkrijk,k=1,2,3 (11)

#### 3.2.2 Objective weight assignment method based on entropy method and improved CRITIC method

Entropy method can reflect the degree of dispersion among sample data, but it can’t show the conflict and difference between indicators. The CRITIC method solves this problem, so this paper comprehensively uses entropy method and improved CRITIC method to calculate the objective weight of indicators [95]. Suppose there are *m* provinces and *n* indexes, which constitute the initial data matrix (*X*_*ij*_)_*m*×*n*_. *X*_*ij*_ represents the value of the j-th index in the i-th province(*i* = 1, 2, ……, *m*: *j* = 1, 2, ……, *n*). Formula (1) is used to standardize the original data, and the normalized matrix is ${\left(X_{ij}^{\text{'}}\right)}_{m\times n}$.

(1) Entropy method
1)Calculate the entropy value of the j-th index *e*_*j*_.
ej=-k∑i=1mpijlnpij (12)

In the above formula, *k* = 1/*ln*(*m*). $P_{ij}=\frac{X_{ij}^{'}}{\sum_{i=1}^{m}X_{ij}^{'}}$.

2) Calculate the weight of the j-th index $W_{j}^{1}$.

$$W_{j}^{1}=\frac{g_{j}}{\sum_{j=1}^{n}g_{j}}$$ (13)

In the above formula, *g*_*j*_ = 1 − *e*_*j*_ represents the difference coefficient of the j-th index.

(2) Improved CRITIC method

CRITIC method is a method of weight calculation first proposed by Diakoulaki in 1995. Its principle is to reflect the objective weight of indicators through the differences and conflicts of indicators [96]. The difference between indicators is reflected by standard deviation, and the conflict is reflected by the value of correlation coefficient. With the further application of this method, researchers found that the conflict between indicators was not related to the positive or negative correlation coefficients [97], and the standard deviation was easily affected by extreme values and had dimensionality. Therefore, this paper improves the CRITIC method by taking absolute value of correlation coefficient and replacing standard deviation with standard deviation coefficient.*C*_*j*_ represents the information content of the j-th index. The larger the value is, the more information content is contained, and the larger the weight $W_{j}^{2}$ of this index is.


Cj=Kj∑i=1n1-rij,j=1,2,⋯,n (14)

Wj2=Cj∑j=1nCj,j=1,2,⋯,n (15)

In formula (14), $K_{j}=\sigma_{j}/\overset{-}{x},\sigma_{j}$ represents the standard deviation of the j-th indicator and *r*_*ij*_ represents the correlation coefficient between the *i* and *j* indicators.

(3) Objective weight *N*_*j*_

The weight $W_{j}^{1}$ and $W_{j}^{2}$ obtained by entropy method and CRITIC method are integrated into objective weight *P*_*j*_. This paper considers that the two objective weighting methods are equally important, so the value of *φ* is selected as 0.5.


Pj=φWj1+1-φWj2 (16)

#### 3.2.3 Combination weighting method based on variance maximization

Suppose a multi-attribute decision making problem has *m* attributes and *n* schemes to be decided. The attribute set is *F* and the scheme set is *X*. *v*_*ij*_ = *f*_*i*_(*x*_*j*_), *i* = 1, 2, ……, *m*, *j* = 1, 2, ……, *n*. Formula (1) is used to standardize the original data, and the decision matrix after normalization of *V* = (*v*_*ij*_)_*m*×*n*_ is *G* = (*g*_*ij*_)_*m*×*n*_. Assume the group FAHP method to calculate the subjective weight is *Q* = (*q*_1_, *q*_2_, ⋯, *q*_*m*_)^*T*^, *$q_{i}\geq 0,\sum_{i=1}^{m}q_{i}=1$*. Integrated objective weight is *P* = (*p*_1_, *p*_2_, ⋯, *p*_*m*_)^*T*^, *$p_{i}\geq 0,\sum_{i=1}^{m}p_{i}=1$*.

To integrate the final weight for *W* = *βQ* + *γP*, including *W* = (*w*_1_, *w*_2_, ⋯, *w*_*m*_)^*T*^, *β*, and *γ* as the linear coefficient of combination weight, and satisfy the unit constraint *β*^2^ + *γ*^2^ = 1(*β* ≥ 0, *γ* ≥ 0). The combination weight of the index can be obtained by calculating the value of *β* and *γ* with the idea of variance maximization.

The idea of variance maximization is to maximize the total variance of all attributes in the attribute set for each scheme when the weight vector is determined. Based on the above analysis, a linear programming model can be established:

maxZ=∑i=1m∑j=1ngij-gij-2wi=∑i=1m∑j=1ngij-gij-2(βqi+γpi) (17)

s.t.β2+γ2=1,β>0,γ>0 (18)

In the above linear programming model, $\overset{-}{g_{ij}}=\frac{1}{n}\sum_{j=1}^{n}g_{ij}$, represents the average value of *n* attributes of the ith attribute.

The solution process of the linear programming equation can be solved by introducing Lagrange function, and the values of *β* and *γ* can be obtained, as shown in Eqs (19) and (20).


β=1/1+∑i=1m∑j=1ngij-gij-2qi∑i=1m∑j=1ngij-gij-2pi (19)

γ=1/1+∑i=1m∑j=1ngij-gij-2pi∑i=1m∑j=1ngij-gij-2qi (20)

In *β* and *γ* value of the known conditions, the combination weights *W* = *βQ* + *γP* can be calculated. But at the moment, *W* = (*w*_1_, *w*_2_, ⋯, *w*_*m*_)^*T*^ not satisfy a necessary condition for $\sum_{i=1}^{m}w_{i}=1$. Therefore, the combination weight *W*_*z*_ = (*W*_*z*1_, *W*_*z*2_, ⋯, *W*_*zm*_)^*T*^ should be normalized.

### 3.3 Calculation of digital economy composite index based on the improved VIKOR method

The VIKOR method is a multi-criteria optimization compromise decision-making method to solve the problem of multi-criteria. It was proposed by Serafim Opricovic in 1995 [98] and has been recognized by researchers at home and abroad since 2004. Based on the maximization of "group benefit" and the minimization of "individual regret value", this method calculates the proximity of each scheme to the ideal point to get the compromise solution [99,100] **错误!未找到引用源.**. In the process of solving the traditional multi-criteria compromise solution, two limiting conditions must be met, that is, the dominant threshold and the acceptability of decision reliability must be considered, which will cause the loss of important information, thus affecting the decision results. In addition, in VIKOR method, the relative distance from the ideal solution is calculated by linear weighting, so the calculated result is greater than the distance calculated by Euclidean distance in TOPSIS method. Large deviation will affect the final sorting result. In order to deeply mine data information and optimize decision results, this paper made two improvements to the traditional VIKOR method: (1) in order to reduce the decision result deviation, Euclidean distance was used instead of linear weighting method to calculate the group utility value; (2) In order to make full use of the inherent information and trend of all the data, grey correlation degree is added into the calculation of the interest ratio of each province to improve the accuracy and observability of the decision results. This article third chapter integrated weight combination empowerment get *W*_*z*_ = (*W*_*z*1_, *W*_*z*2_, ⋯, *W*_*zm*_)^*T*^, assumes that the standardization of the matrix to objectively $X^{\prime }={\left(X_{ij}^{\text{'}}\right)}_{m\times n}$ for *B* = (*b*_*ij*_)_*m*×*n*_. The detailed solution steps are as follows:

(1) Calculate positive and negative ideal solutions *B*^+^ and *B*^−^.
B+=b1+,b2+,⋯,bj+,⋯,bn+,B-=b1-,b2-,⋯,bj-,⋯,bn- (21)
b+=max1≤i≤nbij|j=I,min1≤i≤nbij|j=O,b-=max1≤i≤nbij|j=O,min1≤i≤nbij|j=I (22)

In the above formula, *I* represents the set of efficiency indicators, *O* represents the set of cost indicators.

(2) calculating the provinces groups benefit $S_{i}^{*}$ and individual regret $R_{i}^{*}$, maximum and minimum overall benefits $S_{1}^{*-},S_{1}^{*}$ and individual regret $R_{1}^{*-}$, $R_{1}^{*}$.

Si*=∑j=1nWzjbj+-bij2/bj+-bj-2,i=1,2,⋯,m;j=1,2,⋯,n (23)

Ri*=maxjWzjbj+-bij/bj+-bj-,i=1,2,⋯,m;j=1,2,⋯,n (24)

S1*=miniSi*,S1*-=maxiSi*,i=1,2,⋯,m (25)

R1*=miniRi*,R1*-=maxiRi*,i=1,2,⋯,m (26)

(3) Calculate the grey correlation coefficient $\varepsilon_{ij}^{*}$ and $\varepsilon_{ij}^{-}$ between each province and positive and negative ideal schemes *B*^+^ and *B*^−^. *ρ* is the resolution coefficient, and the value in this paper is 0.5.

εij*=miniminjWzjbij-bj++ρmaximaxjWzjbij-bj+Wzjbij-bj++ρmaximaxjWzjbij-bj+,i=1,2,⋯,m;j=1,2,⋯,n (27)

εij-=miniminjWzjbij-bj-+ρmaximaxjWzjbij-bj-Wzjbij-bj-+ρmaximaxjWzjbij-bj-,i=1,2,⋯,m;j=1,2,⋯,n (28)

(4) Calculate the grey correlation degree $\varepsilon_{i}^{*}$ and $\varepsilon_{i}^{-}$ between each province and positive and negative ideal scheme *B*^+^ and *B*^−^.

εi*=1n∑j=1nεij*,εi-=1n∑j=1nεij-,i=1,2,⋯,m (29)

(5) Based on grey correlation analysis, group utility *S*_*i*_ and individual regret *R*_*i*_, maximum and minimum group utility *S*^−^ and S* and individual regret *R*^−^ and R* were calculated for each province.
Si=εi-εi*,i=1,2,⋯,m (30)
Ri=maxjεi-εij*,i=1,2,⋯,m;j=1,2,⋯,n (31)
S*=miniSi,S-=maxiSi,i=1,2,⋯,m (32)
R*=miniRi,R-=maxiRi,i=1,2,⋯,m (33)
(6) Calculate the interest ratio *H*_*i*_ of each province.
Hi=cVi-V*V--V*+1-cTi-T*T--T*,i=1,2,⋯,m (34)

In the above formula, $V_{i}=S_{i}^{*}*S_{i}$, $T_{i}=R_{i}^{*}*R_{i}$ represents the group utility and individual regret value of the ith province calculated by VIKOR method of grey correlation analysis. $V^{*}=S_{1}^{*}*S^{*}S_{i}$, $V^{-}=S_{1}^{*-}*S^{-},T^{*}=R_{1}^{*}*R^{*}S_{i}$, $T^{-}=R_{1}^{*-}*R^{-}$ represent the improved VIKOR method to calculate the minimum and maximum overall benefit and individual regret. *c* represents the coefficient of decision-making mechanism. If *c* > 0.5, decision-makers tend to prefer provinces with good group benefits. if *c* = 0.5, decision-makers tend to compromise provinces. If *c* < 0.5, decision makers tend to favor provinces with small individual deviations. Therefore, the purpose of setting *c* = 0.5 is to consider both the overall benefit and individual regret value and select a compromise scheme, which is in line with the actual situation. The smaller the profit ratio, the higher the level of digital economy development of each province.

## 4. Empirical analysis

### 4.1 Comprehensive evaluation of the development level of China’s provincial digital economy

#### 4.1.1 Indicators of screening

1. Building a scoring matrix

After the audition index and preliminary screening index. The third part has constructed the digital economy indicator system as shown in Table 1. In order to further optimize the system, this paper uses the method described in Chapter 3 to achieve further screening of indicators. The third-level indicators *B*_21_ ~ *B*_27_ in Table 1 were taken as examples for expert scoring, and the screening process of the remaining indicators was the same. The sample data of evaluation indicators in this paper are obtained by scoring by 6 experts. The scoring data of *B*_21_ ~ *B*_27_ by 6 experts (*y*1 ~ *y*6) are summarized in Table 3.

**Table 3:** Initial expert scoring data.

indicator	*B*_21_	*B*_22_	*B*_23_	*B*_24_	*B*_25_	*B*_26_	*B*_27_
*y*_1_	89	71	88.5	90	82	70	92
*y*_2_	67	70	60	72	84	86	84
*y*_3_	79	94	95	93	85	83.5	82
*y*_4_	76	81	84	80	91	69	79
*y*_5_	86	82	84	66	81	73	95
*y*_6_	77	95	93	67	83	84	93

2. Grey dynamic clustering analysis

According to Formula (1), the initial expert scoring data in Table 3 is standardized, and the standardized data is shown in Table 4.

**Table 4:** Standardized expert scoring data.

indicator	*B*_21_	*B*_22_	*B*_23_	*B*_24_	*B*_25_	*B*_26_	*B*_27_
*y*_1_	1.00	0.04	0.81	0.89	0.10	0.06	0.81
*y*_2_	0.00	0.00	0.00	0.22	0.30	1.00	0.31
*y*_3_	0.55	0.96	1.00	1.00	0.40	0.85	0.19
*y*_4_	0.41	0.44	0.69	0.52	1.00	0.00	0.00
*y*_5_	0.86	0.48	0.69	0.00	0.00	0.24	1.00
*y*_6_	0.45	1.00	0.94	0.04	0.20	0.88	0.88

Grey dynamic clustering is performed on the above data. The grey correlation matrix *C* of the original data set can be calculated by formula (2) and Formula (3) in Chapter 3, and then the fuzzy equivalence relation *C*′ can be obtained by using the transitive closure method.


$$C^{\prime }=\left[\begin{array}{cccccc} 1.000 & 0.5781 & 0.6545 & 0.6125 & 0.6818 & 0.6545\\ & 1.000 & 0.5781 & 0.5781 & 0.5781 & 0.5781\\ & & 1.000 & 0.6125 & 0.6545 & 0.7273\\ & & & 1.000 & 0.6125 & 0.6125\\ & & & & 1.000 & 0.6545\\ & & & & & 1.000 \end{array}\right]$$

Different *λ* values correspond to different classification results. The *λ* intercept matrix is used for clustering, and the largest element in matrix *C*′ is taken successively. The clustering results are shown in Table 5.

**Table 5:** Clustering results of the original matrix.

*λ*	Classification number	The classification results
1	6	{*y*_1_}, {*y*_2_}, {*y*_3_}, {*y*_4_}, {*y*_5_}, {*y*_6_}
0.7273	5	{*y*_1_}, {*y*_2_}, {*y*_3_, *y*_6_}, {*y*_4_}, {*y*_5_}
0.6818	4	{*y*_1_, *y*_5_}, {*y*_2_}, {*y*_4_}, {*y*_3_, *y*_6_}
0.6545	3	{*y*_1_, *y*_3_, *y*_5_, *y*_6_}, {*y*_4_}, {*y*_2_}
0.6125	2	{*y*_1_, *y*_3_, *y*_4_, *y*_5_, *y*_6_}, {*y*_2_}
0.5781	1	{*y*_1_, *y*_2_, *y*_3_, *y*_4_, *y*_5_, *y*_6_}

Cluster types affect dynamic clustering results. In order to obtain more scientific clustering results, it is necessary to seek the classification corresponding to the optimal threshold. The F-statistic contains the distance between every two categories, and the category with the maximum F-statistic corresponds to the optimal threshold *λ*. In this paper, Formula (4) is used to calculate F values of various categories, as shown in Table 6. Since the classification number of 1 and 8 is meaningless to the dynamic clustering process, these two cases are not discussed. As can be seen from Table 6, when the F-statistic achieves the maximum value, the corresponding classification number is 4, the optimal threshold *λ* value is 0.6818, and the optimal classification is *U*⁄*B*_2_ = {{*y*1, *y*5}, {*y*2}, {*y*4}, {*y*3, *y*6}}.

**Table 6:** F- Statistical scale.

Classification number	2	3	4	5
*λ*	0.6125	0.6545	0.6818	0.7273
F	1.3657	1.4614	2.2983	1.6993

3. Index screening based on rough set

The process of rough intensive reduction is to remove one indicator each time to conduct grey clustering, find the best clustering result, compare it with the original clustering result, and delete the indicator that has no influence on the clustering result. In this study, the whole sample set consists of 6 senior experts. Each indicator of digital economy represents conditional attribute set *C*, and the sample clustering result represents decision attribute set *D*. In this paper, *B*_21_ ~ *B*_27_ were successively deleted for gray dynamic clustering. The clustering results are shown in Table 7.

**Table 7:** Clustering results.

serial number	Indicators	Clustering results
1	All indicators C	{*y*_1_, *y*_5_}, {*y*_2_}, {*y*_4_}, {*y*_3_, *y*_6_}
2	*B*_2_ − *B*_21_	{*y*_1_}, {*y*_2_}, {*y*_3_, *y*_6_}, {*y*_4_}, {*y*_5_}
3	*B*_2_ − *B*_22_	{*y*_1_, *y*_5_}, {*y*_2_}, {*y*_3_}, {*y*_4_}, {*y*_6_}
4	*B*_2_ − *B*_23_	{*y*_1_}, {*y*_2_}, {*y*_3_, *y*_6_}, {*y*_4_}, {*y*_5_}
5	*B*_2_ − *B*_24_	{*y*_1_}, {*y*_2_}, {*y*_3_, *y*_6_}, {*y*_4_}, {*y*_5_}
6	*B*_2_ − *B*_25_	{*y*_1_, *y*_3_, *y*_4_, *y*_5_, *y*_6_}, {*y*_2_}
7	*B*_2_ − *B*_26_	{*y*_1_, *y*_3_, *y*_4_, *y*_5_, *y*_6_}, {*y*_2_}
8	*B*_2_ − *B*_27_	{*y*_1_, *y*_5_}, {*y*_2_}, {*y*_4_}, {*y*_3_, *y*_6_}

According to the dynamic clustering results in Table 7, *IND*(*B*_2_) = *IND*(*B*_2_ − {*B*_27_}). The optimal clustering result after removing indicator *B*_27_ is equivalent to the clustering result under all samples. This indicator has no influence on the comprehensive evaluation result, so index *B*_27_ is deleted. In the same way as the above analysis, this paper carries out grey dynamic clustering and index reduction for the remaining indicators, and the results are marked in the last column of Table 1. In this way, the original indicators are condensed into 36 indicators, and the specific reduction results are presented in Table 8. Table 8 is the code representation of the indicator system after indicator reduction.

**Table 8:** Index weight of digital economy index system.

First-Level Index	*Q* ^1^	Second-Level Index	*Q* ^2^	Third-Level Index	$Q_{j}^{3}$	*Q*_*j*_	$W_{j}^{1}$	$W_{j}^{2}$	*P*_*j*_	*W*_*zj*_
A	0.3022	*A*_1_	0.2854	*A*_11_	0.3389	0.0292	0.0290	0.0338	0.0291	0.0292
				*A*_12_	0.3445	0.0297	0.0274	0.0268	0.0285	0.0291
				*A*_13_	0.3166	0.0273	0.0282	0.0245	0.0277	0.0275
		*A*_2_	0.2627	*A*_21_	0.2887	0.0229	0.0275	0.0233	0.0252	0.0241
				*A*_22_	0.2441	0.0194	0.0277	0.0239	0.0235	0.0214
				*A*_23_	0.2599	0.0206	0.0271	0.0265	0.0239	0.0223
				*A*_24_	0.2073	0.0165	0.0274	0.0289	0.0219	0.0192
		*A*_3_	0.2217	*A*_31_	-	0.0670	0.0269	0.0212	0.0470	0.0570
		*A*_4_	0.2302	*A*_41_	0.4833	0.0336	0.0269	0.0257	0.0303	0.0320
				*A*_42_	0.5167	0.0359	0.0306	0.0274	0.0333	0.0346
B	0.2739	*B*_1_	0.4333	*B*_11_	0.3778	0.0448	0.0274	0.0310	0.0361	0.0405
				*B*_12_	0.3444	0.0409	0.0286	0.0212	0.0347	0.0378
				*B*_13_	0.2778	0.0330	0.0261	0.0266	0.0295	0.0312
		*B*_2_	0.5667	*B*_21_	0.174281	0.0271	0.0280	0.0317	0.0275	0.0273
				*B*_22_	0.146066	0.0227	0.0288	0.0344	0.0257	0.0242
				*B*_23_	0.168723	0.0262	0.0300	0.0259	0.0281	0.0271
				*B*_24_	0.195077	0.0303	0.0274	0.0282	0.0289	0.0296
				*B*_25_	0.168959	0.0262	0.0271	0.0233	0.0267	0.0265
				*B*_26_	0.146795	0.0228	0.0250	0.0516	0.0239	0.0233
C	0.1922	*C*_1_	0.3722	*C*_11_	-	0.0715	0.0280	0.0269	0.0498	0.0606
		*C*_2_	0.3334	*C*_21_	0.3833	0.0246	0.0267	0.0245	0.0256	0.0251
				*C*_22_	0.6167	0.0395	0.0332	0.0379	0.0364	0.0380
		*C*_3_	0.2944	*C*_31_	0.3389	0.0192	0.0271	0.0190	0.0231	0.0211
				*C*_32_	0.3167	0.0179	0.0269	0.0308	0.0224	0.0202
				*C*_33_	0.3444	0.0195	0.0273	0.0336	0.0234	0.0214
D	0.2317	*D*_1_	0.35	*D*_11_	0.6333	0.0514	0.0286	0.0254	0.0400	0.0457
				*D*_12_	0.3667	0.0297	0.0266	0.0323	0.0282	0.0290
		*D*_2_	0.3278	*D*_21_	0.2119	0.0161	0.0279	0.0297	0.0220	0.0191
				*D*_22_	0.1481	0.0112	0.0271	0.0213	0.0192	0.0152
				*D*_23_	0.2179	0.0165	0.0258	0.0266	0.0212	0.0189
				*D*_24_	0.2252	0.0171	0.0274	0.0271	0.0222	0.0197
				*D*_25_	0.1969	0.0150	0.0269	0.0299	0.0209	0.0179
		*D*_3_	0.3222	*D*_31_	0.2884	0.0215	0.0308	0.0219	0.0262	0.0239
				*D*_32_	0.2251	0.0168	0.0274	0.0255	0.0221	0.0195
				*D*_34_	0.3022	0.0226	0.0281	0.0275	0.0253	0.0239
				*D*_35_	0.1843	0.0138	0.0275	0.0245	0.0206	0.0172

#### 4.1.2 comprehensive assessment

1. Determine the weight of evaluation index system

This paper uses the group FAHP method introduced in Chapter 3 to calculate the subjective weight of indicators. In the same way as the index screening process, three experts were invited to score the third-level indexes *B*_21_ ~ *B*_27_ by referring to the rules in Table 2. The three experts give the initial fuzzy judgment matrix according to the relative importance of the third-level index compared with the upper-level index *B*_2_. The judgment matrices provided by the three experts after consistency test and adjustment are $R_{B}^{\left(s1\right)}$, $R_{B}^{\left(s2\right)}$ and $R_{B}^{\left(s3\right)}$, as shown in Tables 9–11.

**Table 9:** Judgment matrix $R_{B}^{\left(s1\right)}$ of expert *s*1.

*B*_2_	*B*_21_	*B*_21_	*B*_21_	*B*_21_	*B*_21_	*B*_21_	weight	$d_{B}^{\left(s1\right)}$
*B*_21_	0.50	0.60	0.60	0.40	0.55	0.55	0.18	0.095
*B*_22_	0.40	0.50	0.45	0.30	0.30	0.45	0.127	
*B*_23_	0.40	0.55	0.50	0.45	0.55	0.60	0.17	
*B*_24_	0.60	0.70	0.55	0.50	0.70	0.75	0.22	
*B*_25_	0.45	0.70	0.45	0.30	0.50	0.60	0.167	
*B*_26_	0.45	0.55	0.40	0.25	0.40	0.50	0.137	

**Table 10:** Judgment matrix $R_{B}^{\left(s2\right)}$ of expert *s*2.

*B*_2_	*B*_21_	*B*_21_	*B*_21_	*B*_21_	*B*_21_	*B*_21_	weight	$d_{B}^{\left(s2\right)}$
*B*_21_	0.50	0.55	0.45	0.45	0.50	0.60	0.170	0.08
*B*_22_	0.45	0.50	0.60	0.40	0.40	0.60	0.163	
*B*_23_	0.55	0.40	0.50	0.45	0.45	0.50	0.157	
*B*_24_	0.55	0.60	0.55	0.50	0.60	0.60	0.193	
*B*_25_	0.50	0.60	0.55	0.40	0.50	0.55	0.173	
*B*_26_	0.40	0.40	0.50	0.40	0.45	0.50	0.143	

**Table 11:** Judgment matrix $R_{B}^{\left(s3\right)}$ of expert *s*3.

*B*_2_	*B*_21_	*B*_21_	*B*_21_	*B*_21_	*B*_21_	*B*_21_	weight	$d_{B}^{\left(s3\right)}$
*B*_21_	0.50	0.60	0.45	0.55	0.55	0.45	0.173	0.085
*B*_22_	0.40	0.50	0.40	0.45	0.50	0.45	0.147	
*B*_23_	0.55	0.60	0.50	0.55	0.45	0.55	0.180	
*B*_24_	0.45	0.55	0.45	0.50	0.60	0.55	0.173	
*B*_25_	0.45	0.50	0.55	0.40	0.50	0.60	0.167	
*B*_26_	0.55	0.55	0.45	0.45	0.40	0.50	0.160	

The consistency index $d_{B}^{\left(s1\right)}$, $d_{B}^{\left(s2\right)}$ and $d_{B}^{\left(s3\right)}$ of the judgment matrix are calculated according to Formula (6) in Chapter 3, and then the objective weight vector of experts is calculated according to Formula (9). Since the subjective weight of experts is *ϑ* = (0.333, 0.333, 0.333), the weight vector *M*_*k*_ = (0.318, 0.346, 0.336) of experts can be obtained from Eq (10). This article assumes that the consistency index of the critical value Y is 0.1, $d_{B}^{\left(s1\right)}$, $d_{B}^{\left(s2\right)}$, $d_{B}^{\left(s3\right)}$ are less than 0.1, so the matrix $R_{B}^{\left(s1\right)}$, $R_{B}^{\left(s2\right)}$, $R_{B}^{\left(s3\right)}$ is a consistent matrix. Therefore, the satisfaction agreement matrix of these three experts can be integrated into a comprehensive judgment matrix $\overset{-}{R_{B2}}$ (as shown in Table 12) by Eq (11). The matrix is also a satisfaction agreement matrix, so there is no need to conduct consistency test.

**Table 12:** Comprehensive judgment matrix (R_B2) 12.

*B*_2_	*B*_21_	*B*_21_	*B*_21_	*B*_21_	*B*_21_	*B*_21_	weight
*B*_21_	0.5000	0.5826	0.4976	0.4676	0.5326	0.5337	0.1743
*B*_22_	0.4173	0.5000	0.4851	0.3850	0.4017	0.5019	0.1461
*B*_23_	0.5023	0.5148	0.5000	0.4835	0.4817	0.5485	0.1687
*B*_24_	0.5323	0.6149	0.5164	0.5000	0.6317	0.6308	0.1951
*B*_25_	0.4673	0.5982	0.5182	0.3682	0.5000	0.5826	0.1690
*B*_26_	0.4662	0.4980	0.4514	0.3691	0.4173	0.5000	0.1468

The weights in Table 12 are the expert weights of indicators *B*_21_ ~ *B*_27_. In the same way as the above steps, the expert weights of the first-level and second-level indicators in the index system in this paper can be obtained. In Table 8, *Q*^1^, *Q*^2^, $Q_{j}^{3}$ and *Qj* represent the expert weight of first-level indicator, second-level indicator, third-level indicator and the final subjective weight of third-level indicator respectively, where $Q_{j}=Q^{1}*Q^{2}*Q_{j}^{3}$.

The objective weight of the index is calculated according to formulas (12) ~ (16) and shown in Table 8. In Table 8, $W_{j}^{1}$ and *$W_{j}^{2}$* represent the index weight calculated by entropy method and improved CRITIC, and *P*_*j*_ represents the final objective weight calculated by integrating the two objective weights.

Using variance maximization to synthesize the subjective and objective weights, *W*_*zj*_ is calculated from Eqs (17) ~ (20), where *β* = 0.7053 and *γ* = 0.7089 of the integrated weights are expressed in Table 8.

2. Comprehensive evaluation based on improved VIKOR method

The comprehensive weight of the indicators has been calculated. According to the improved VIKOR model given in Chapter 3, the digital economy development level of all indicators is measured, and the interest ratio *H*_*i*_ of the digital economy development level of 31 Provinces in China from 2013 to 2019 is obtained, as shown in Table 13. Through the testing process, it can be obtained that the sample results meet the two constraints of the multi-attribute compromise solution method and can be sorted according to the value of the benefit ratio.

**Table 13:** The profit ratio of the level of digital economy development from 2013 to 2019.

Province	2013	2014	2015	2016	2017	2018	2019	Average
Beijing	0.0000	0.0000	0.0123	0.0007	0.0000	0.0012	0.0058	0.0029
Tianjin	0.5351	0.5565	0.5424	0.5462	0.5255	0.4183	0.3432	0.4953
Hebei	0.6400	0.6707	0.6551	0.6511	0.6020	0.4813	0.3518	0.5789
Shanxi	0.6931	0.7257	0.7536	0.7625	0.7447	0.6212	0.4692	0.6815
Inner Mongolia	0.7246	0.7505	0.7766	0.7728	0.7530	0.6546	0.4976	0.7042
Liaoning	0.5836	0.6091	0.6008	0.6331	0.6299	0.5244	0.3967	0.5682
Jinlin	0.6949	0.7059	0.7407	0.7638	0.7342	0.6202	0.4462	0.6723
Lheilongjiang	0.7061	0.7115	0.7554	0.7747	0.7492	0.6487	0.5166	0.6946
Shanghai	0.1138	0.0969	0.1082	0.1155	0.2021	0.1458	0.1096	0.1274
Jiangsu	0.3473	0.3363	0.3115	0.3197	0.3504	0.2121	0.1548	0.2903
Zhejiang	0.2273	0.1619	0.1356	0.1065	0.2368	0.1325	0.3083	0.1870
Anhui	0.6986	0.7461	0.7298	0.6952	0.6448	0.5126	0.5925	0.6599
Fujian	0.5003	0.5025	0.5041	0.5826	0.5060	0.3953	0.5493	0.5057
Jiangxi	0.8178	0.8448	0.8163	0.8271	0.7668	0.6230	0.5963	0.7560
Shandong	0.5580	0.5652	0.5519	0.5279	0.5151	0.4160	0.4408	0.5107
Henan	0.7630	0.7714	0.7783	0.7771	0.7622	0.6147	0.5918	0.7226
Hubei	0.6078	0.6238	0.6160	0.5991	0.5883	0.5261	0.5252	0.5838
Hunan	0.7005	0.7452	0.7558	0.7230	0.6761	0.5776	0.6681	0.6923
Guangdong	0.2051	0.1745	0.1627	0.1098	0.1818	0.2113	0.3004	0.1922
Guangxi	0.8116	0.8208	0.8677	0.8360	0.7799	0.6703	0.7791	0.7951
Hainan	0.8464	0.8519	0.8831	0.8335	0.8259	0.8146	0.8156	0.8387
Chongqing	0.6872	0.6910	0.6696	0.6578	0.6467	0.6527	0.6493	0.6649
Sichuan	0.6187	0.6227	0.6091	0.5959	0.5764	0.5809	0.5791	0.5975
Guizhou	0.8989	0.8897	0.9168	0.8576	0.8095	0.7804	0.7686	0.8459
Yunnan	0.8663	0.8680	0.8641	0.8402	0.8328	0.8172	0.8239	0.8446
Tibet	0.9931	0.9913	0.9700	0.9763	0.9903	0.9632	0.9903	0.9821
Shaanxi	0.6220	0.6151	0.6548	0.6385	0.6218	0.6430	0.6929	0.6412
Gansu	0.8578	0.8833	0.9002	0.9177	0.9434	0.9563	0.9907	0.9213
Qinghai	0.8500	0.8507	0.9023	0.8712	0.8732	0.8213	0.8925	0.8659
Ningxia	0.8221	0.8297	0.8501	0.8382	0.8344	0.7894	0.8415	0.8293
Xinjiang	0.8381	0.8511	0.8717	0.8494	0.8457	0.8793	0.9126	0.8640

### 4.2 Analysis of system measurement results

#### 4.2.1 An overall analysis of the development of China’s provincial digital economy

**Fig 4:**
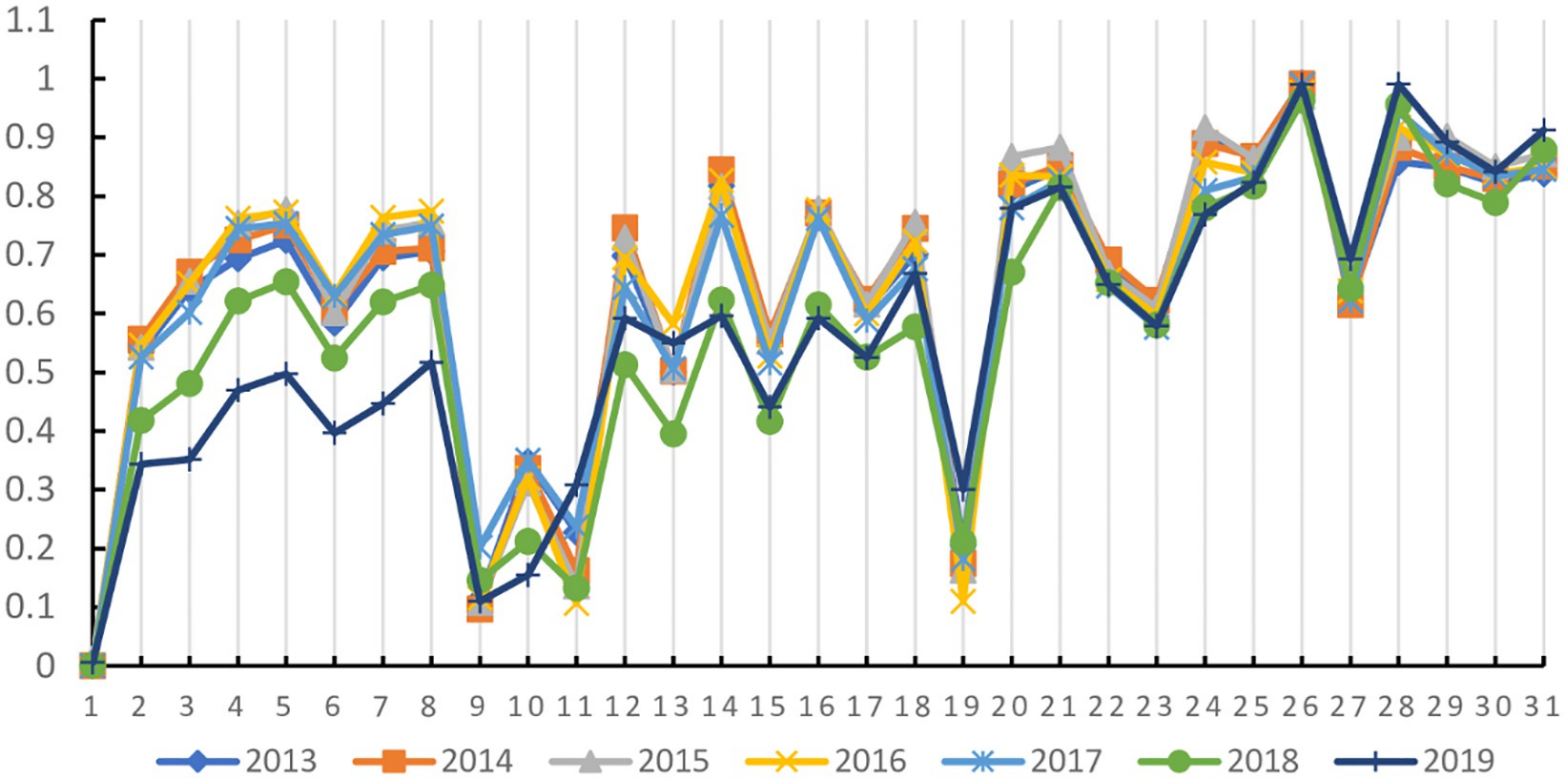
Chart of the changing trend of digital economy development level in China’s provinces from 2013 to 2019.

According to the calculation results in Table 13, the change trend chart of digital economy development of China’s provinces from 2013 to 2019 is made (as shown in Fig 4). The provinces in Fig 4 are represented by numbers 1 to 31 in the order shown in Table 13. The data in Fig 4 are VIKOR values of each province, so the smaller the value, the higher the digital economy level.

As can be seen from Fig 4, the development level of digital economy of all Provinces in China from 2013 to 2019 shows a trend of superposition and increasing year by year, although the status of some years or provinces fluctuates. (1) The development of digital economy in Beijing, Shanghai, Zhejiang, Guangdong and Jiangsu is the leader in the whole country, and Beijing is the leader in the first queue. The average VIKOR value of its digital economy development level from 2013 to 2019 is as low as 0.0029, and even 0 in some years. (2) Gansu and Tibet have the lowest digital economic development among 31 provinces and regions, and belong to the last echelon, with the profit ratio of 0.9213 and 0.9821 respectively. The VIKOR value of Tibet is close to 1, indicating that the development of digital economy is quite backward. (3) The VIKOR value gap of digital economy development in Tianjin, Fujian, Shandong, Liaoning, Hebei, Hubei, Sichuan, Shaanxi, Anhui, Chongqing, Jilin, Shaanxi, Hunan, Heilongjiang, Inner Mongolia, Henan, Jiangxi, Guangxi and other provinces is around 0.3, which can be roughly regarded as provinces at the same level.

#### 4.2.2 Provincial analysis of China’s digital economy development

In view of the above analysis and calculation results, this paper will study the development level and potential of digital economy from different regions, and divide 31 provinces into four major economic regions according to the national classification of economic zones, as shown in Table 14.

**Table 14:** Classification of China’s four major economic regions.

Economic Region	Province
Eastern Region	Beijing, Tianjin, Hebei, Shanghai, Jiangsu, Zhejiang, Fujian, Shandong, Guangdong, Hainan
Middle Region	Shanxi, Anhui, Jiangxi, Henan, Hubei, Hunan
West Region	Inner Mongolia, Guangxi, Chongqing, Sichuan, Guizhou, Yunnan, Tibet, Shaanxi, Gansu, Qinghai, Ningxia, Xinjiang
Northeast Region	Liaoning, Jilin, Heilongjiang

This paper will divide the development level of provincial digital economy into different categories, and the specific classification rules are as follows: When the interest ratio *H*_*i*_ ∈ [0,0.35), the development intensity of digital economy is very strong and marked as *I*; when the interest ratio *H*_*i*_ ∈ [0.35,0.6), the development intensity is strong and marked as *II*; when the interest ratio *H*_*i*_ ∈ [0.6,0.7), the development intensity is general and marked as *III*; When the interest ratio *H*_*i*_ ∈ [0.7,0.85), the development intensity is weak and marked as *IV*; when the interest ratio *H*_*i*_ ∈ [0.85,1) the development intensity is very weak and marked as *V*. According to the above classification rules and the value of the interest ratio in Table 4, the classification of the development level of provincial digital economy from 2013 to 2019 can be obtained, as shown in Table 15.

**Table 15:** Digital economy development level classification from 2013 to 2019.

Classification Code	Classification Strength	Province
I	Very Strong	Beijing, Shanghai, Zhejiang, Guangdong, Jiangsu
II	Strong	Tianjin, Fujian, Shandong, Liaoning, Hebei, Hubei, Sichuan
III	General	Shaanxi, Anhui, Chongqing, Jilin, Shanxi, Hunan, Heilongjiang
IV	Weak	Inner Mongolia, Henan, Jiangxi, Guangxi, Ningxia, Hainan, Guizhou, Yunnan
V	Very Weak	Tibet, Gansu, Qinghai, Xinjiang

Table 15 shows the following:(1) Provinces located in the *I* and *II* gradient belong to the category with good development momentum. Almost all the provinces and cities with rapid development of digital economy come from the eastern region, which has a high degree of openness, high level of scientific and technological innovation, high level of citizens’ education, and fast transformation efficiency of innovation achievements. It is a fertile soil for various emerging economies to take root. (2) Among provinces with average level of digital economy development, Shaanxi and Chongqing are from western China, Jilin and Heilongjiang are from northeast China and the rest are from central China. (3) Except for Henan and Jiangxi, the provinces and cities in the *IV* and *V* grade of digital economy development are all from the west. The economic development of western China is still at the bottom of the whole country.

**Fig 5:**
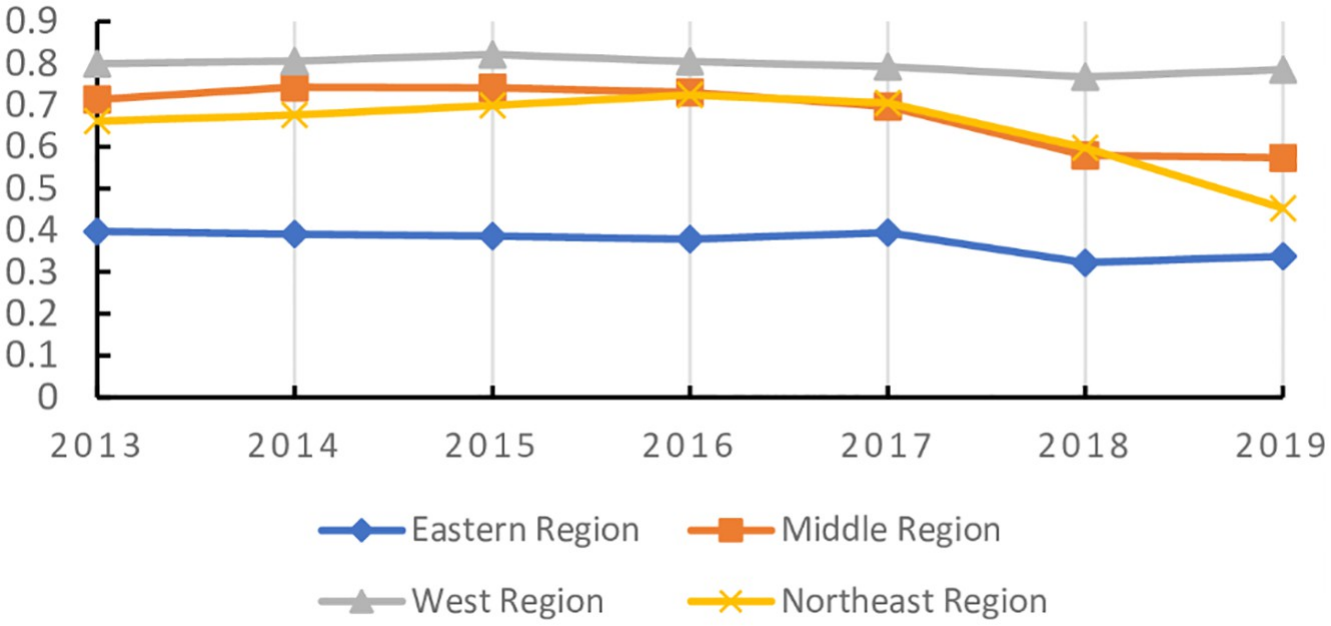
Chart of changes in the level of digital economy in China’s four economic regions from 2013 to 2019.

According to Table 13, we can calculate the average VIKOR value of digital economic development in the eastern, western, central and northeastern regions from 2013 to 2019, and draw a line chart of the changing state in Fig 5. As can be seen from Fig 5, the western region has the highest average interest ratio, so its digital economy development capacity is the weakest and has been stagnant, stably remaining at the bottom of the country; The average interest ratio of central region and northeast region was particularly high at the beginning. Although it rose in the middle, both regions gradually declined significantly in 2019, presenting a trend of "high open and low VIKOR value", even leveling and crossing. This shows that the development of digital economy gradually tends to be clear. The average profit ratio of the eastern region is the lowest in China, and the development of digital economy is far ahead.

**Fig 6:**
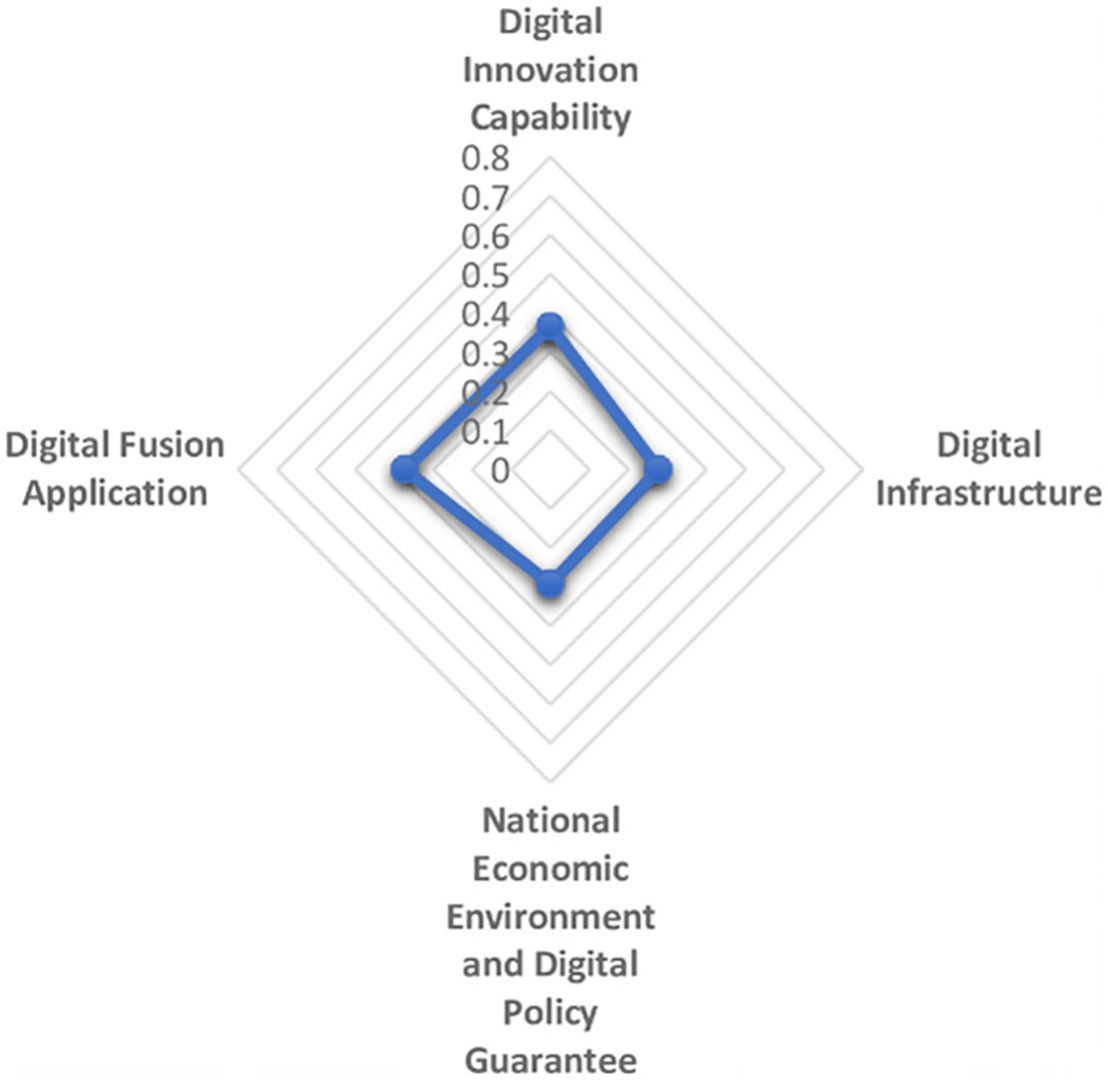
Radar map of eastern digital economy development.

**Fig 7:**
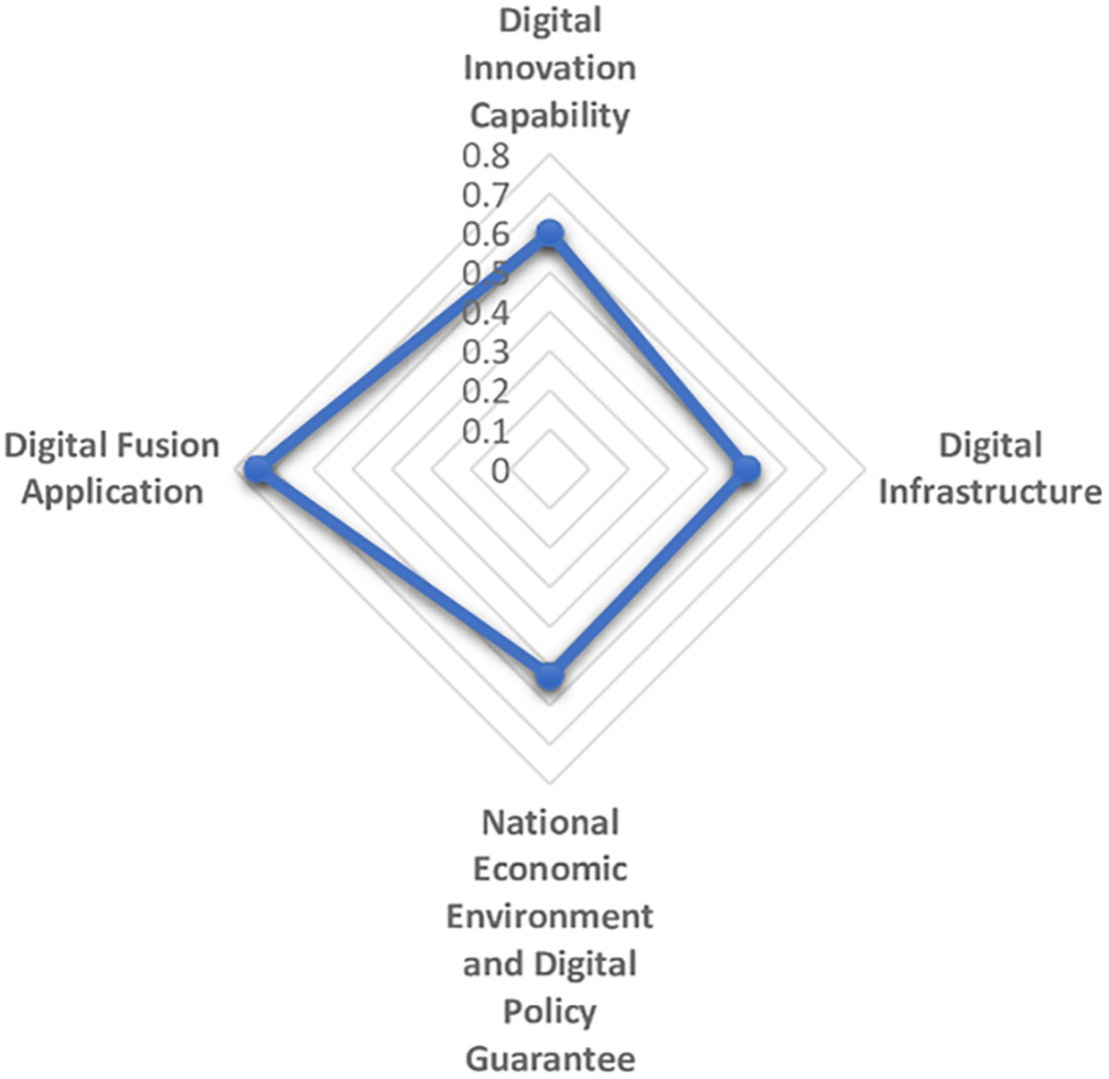
Radar map of digital economy development in central China.

**Fig 8:**
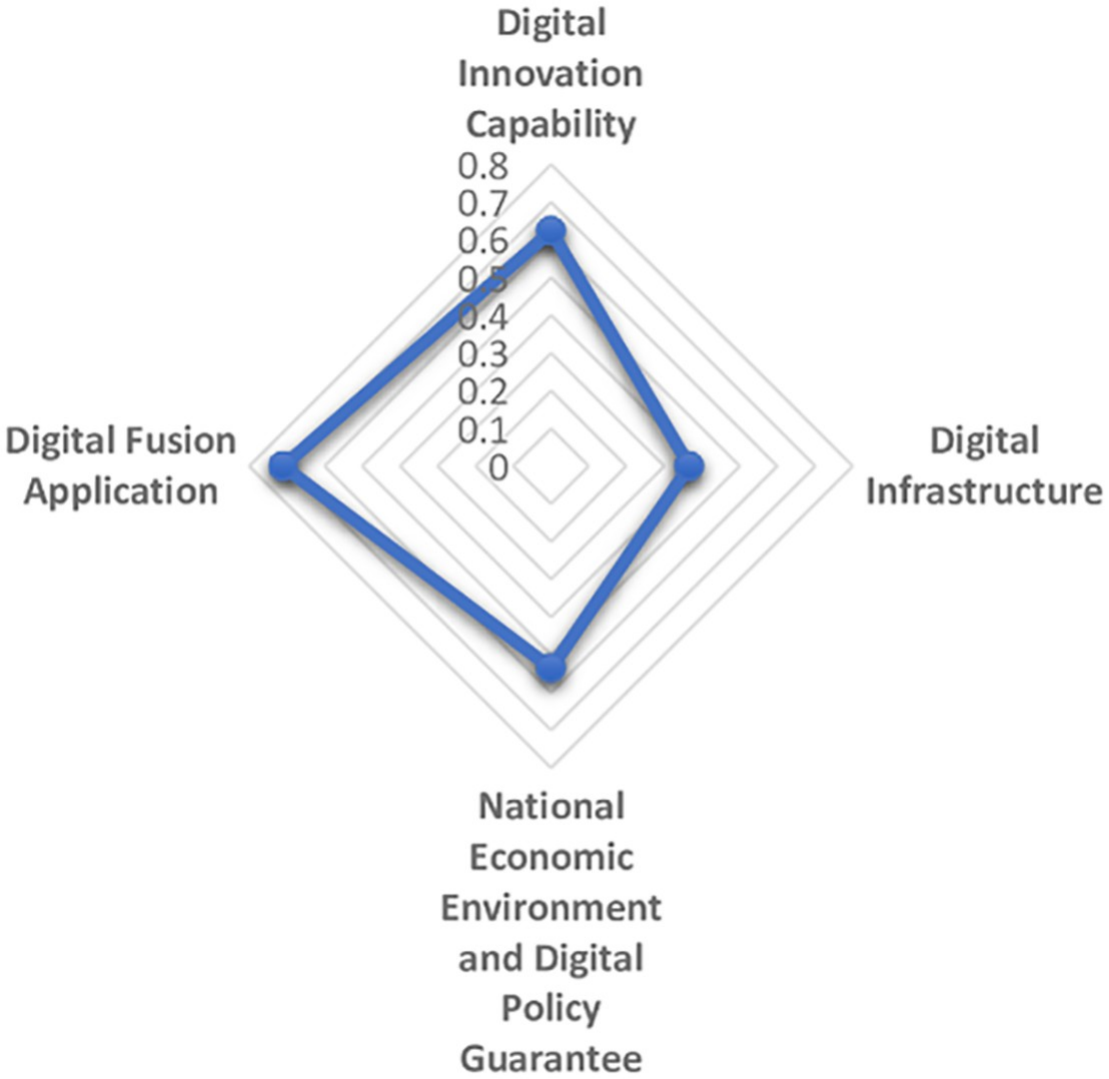
Radar map of northeast digital economy development.

**Fig 9:**
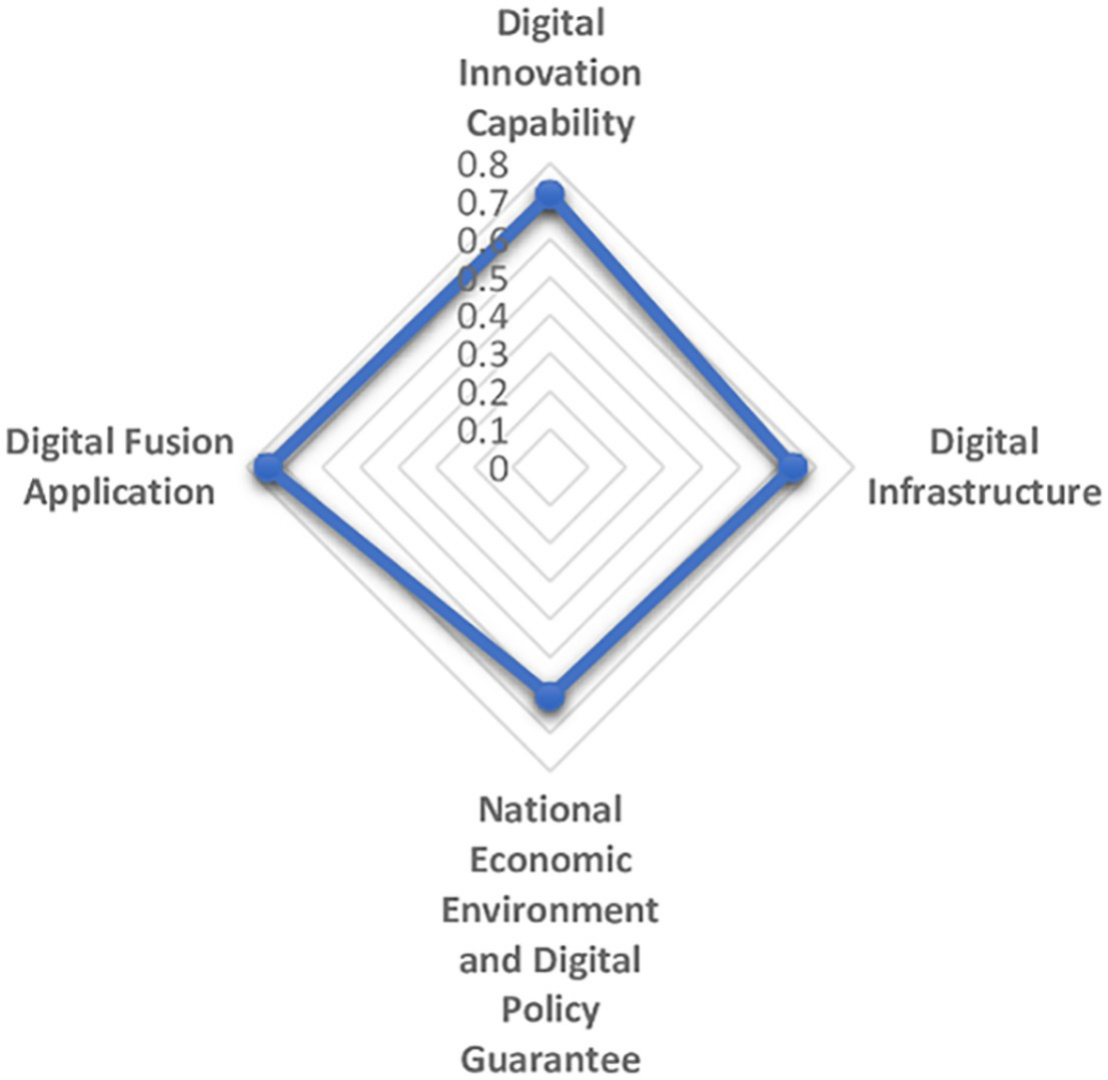
Radar map of western digital economy development.

In combination with the interest ratio value of digital economy development level of each province, this paper divides the digital economy development level of China’s four economic regions into four dimensions of first-level indicators for comparative analysis, as shown in Figs 6–9.

Provinces in the eastern economic zone are all developed coastal areas, and the average profit ratio of digital economic development has been stable at about 0.4 in the past seven years, ranking first among the four major economic regions. The two dimensions of economic development environment and policy system guarantee, digital infrastructure construction capacity are slightly ahead of digital innovation support and digital integration application. Beijing and Shanghai are in a strong position in the development of the region and even the whole country, while Zhejiang, Guangdong and Jiangsu are slightly inferior to Shanghai and Beijing in the development of digital economy. But overall, the gap is small.

The central and northeast economic zones are regions with strong development potential. The advantages of the two economic zones are obvious and their weaknesses are prominent, but they are both in a better situation and have been improved every year. Compared with the other two dimensions, the economic development environment and digital policy system guarantee, digital infrastructure construction capacity of the central economic zone are stronger, but far inferior to developed provinces, belonging to a relatively low level. The situation in the northeast is similar to the central region, but the infrastructure capacity of the northeast is slightly better than that of the central region. The main reason for this is that the three northeastern provinces have long been China’s heavy industry development base and have the technology and ability to build digital infrastructure. The western economic zone is a relatively backward region, which is at the bottom of the development of digital economy in China. The development capacity of each dimension is not high, and the reason why economic environment and policy system guarantee are better than the other three dimensions mainly comes from the preferential policies and systems of the state to the western backward regions. The western region lags behind the country in terms of openness and innovation, infrastructure construction, business environment, attracting foreign investment and human resources.

To sum up, except for the western economic zone, the other three economic zones have significant advantages in the development of digital economy, but obvious shortcomings. There are significant differences in resource endowment, geographical location, industrial structure and innovation capacity. Therefore, the development level of digital economy appears regional characteristics, and the gap is larger. Under the background of constructing the domestic and international double circulation development pattern and vigorously promoting the construction of digital China, the government should focus on the development of backward regions and carry out preferential policies. Under the condition of actively implementing the strategy of western development and the rise of the central region, the economy of the northeast region and the economy of the east region will be revitalized by a new leap, and the gap between the development of digital economy in developed regions and backward regions will be narrowed. In addition, digital economic development at the general level of the region should break the bottleneck, to achieve a new leap.

## 5. Conclusion

### 5.1 Discussion

In order to reflect the essence of digital economy and comprehensively reflect the development of digital economy, this paper starts from relevant literature research and combines the whole process of digital economy development. The process of digital input to digital output is revealed by incorporating digital security capability into the index system. The index system is constructed from four aspects: digital innovation support, infrastructure support, national economic environment and policy guarantee, and digital integration and application. In this paper, sample data of Chinese provinces from 2013 to 2019 are selected. Firstly, an indicator system of digital economy is preliminarily constructed through indicator screening based on grey dynamic clustering and rough intensive reduction theory. Then, group FAHP method is used to calculate the subjective weight, and entropy method and improved CRITIC method are used to calculate the objective weight. The subjective and objective weights are integrated by the idea of variance maximization. Finally, grey relational analysis is combined with the improved VIKOR method to establish a comprehensive evaluation model of digital economy development level. The research results in this paper are consistent with the existing research results using different measurement methods [28], which proves the reliability and validity of the research in this paper. Specific research results are as follows:

1. As a whole, the development capacity of China’s provincial digital economy is improving year by year. Although there have been some fluctuations in some years, the overall trend for the better is beyond doubt. The eastern economic zone of the coastal provinces is the locomotive and bellwether of the development of the national digital economy. The development capacity of the central economic zone is in the middle position, followed by the northeastern economic zone and the western economic zone at the bottom. Among them, the average interest ratio of digital economy development in northeast and central economic zones shows a phenomenon of "high opening and low trend", with great development potential.
2. On the whole, China’s provincial digital infrastructure construction capacity, economic environment and policy guarantee capacity are slightly higher than the other two dimensions. From the regional perspective, the development level of all dimensions of the eastern economic zone is in a leading position, but relatively speaking, the biggest weakness lies in the application of digital integration. The biggest weakness of the central and northeast economic zones is digital application, followed by digital innovation ability. Among them, the digital infrastructure construction capacity of northeast China is better than that of central Economic zone, but it still belongs to low level gradient. The development level of all dimensions in western backward regions is weak.

### 5.2 Implications

According to the above research results, it is necessary to realize the high-quality development of digital economy and build a new development pattern of double circulation. In pursuit of the goal of green, sustainable and circular economic development, this paper puts forward the following suggestions:

1. Optimize the industrial layout of digital economy according to local conditions in view of unbalanced regional development. Each region has its own unique advantages in resource endowment, geographical location, natural conditions and other characteristics, resulting in the development level of digital economy there is a large gap between the phenomenon of regional development. In order to narrow regional differences, first of all, it is necessary to predict in advance the "Digital Divide" between the east and the west in China’s digital economy development, exert the government’s macro-control function to the maximum extent, gather limited capital, talent and technology in the western region, and formulate policies and guarantee systems with their own characteristics for each region. Secondly, the government should vigorously boost the aggregation effect and diffusion effect of the development of digital economy in coastal developed areas. In addition, in order to achieve the national situation of "point to area, from line to area, and gradually form regional cooperation", the government should further exert the radiation effect of regional ranking to the maximum. Finally, the paper proposes the layout of digital economy development in accordance with the actual situation of each province or region, and creates digital economy industry gathering area, innovation area and experimental area with its own advantages in each province or region. In this way, the development level of digital economy in underdeveloped and backward regions can be improved, the "Matthew effect" can be broken, and the development gap between provinces in China’s digital economy can be narrowed.
2. Enhance the support for digital innovation and infrastructure construction, and build a solid input end of the digital economy. On the one hand, for improving digital innovation, increasing the input of digital talents, capital, data elements and technology is equivalent to accumulating the innovation capacity needed by the development of digital economy. Northeast and Midwest economic zones should increase investment in digital economy-related industries, establish large databases and cloud networks containing data elements of national nature, and cultivate talents with new digital knowledge and high skills in line with the development of digital economy through school-enterprise cooperation. At the same time, local governments should take digitalization as the leading role, implement innovation-driven development strategy, and strengthen the leading role of technological innovation. On the other hand, in order to improve the capacity of new infrastructure construction, we should further increase R&D investment in new information and communication technologies and key core areas, encourage enterprises and individuals to carry out technological innovation in key areas, break through core technologies, and achieve self-sufficiency in core components and basic materials. For provinces and cities along the eastern coast, they should focus on the construction of new infrastructure such as IPV6, 5G base stations and artificial intelligence to strengthen their leading position and carry out technology spillover and diffusion to other regions. Provinces and cities in central and western China and northeast China should implement simultaneous investment in traditional and new infrastructure, focusing on the construction of new infrastructure, and strive to quickly break the obstacles in infrastructure construction.
3. Deepen the application of digital integration and enrich the output end of the digital economy.

The integration and application of digital technology is the ultimate goal of the development of digital economy, and it is the necessary premise to build a double circular economy to get through the combination of digital technology and three industries. On the one hand, the country should integrate existing digital economy platforms and build a national integrated service platform. This approach can reduce the cost of the platform application and provide each customer with a customized precise digital plan. On the other hand, industrial departments and enterprises should be encouraged to increase in-depth cooperation with research institutes and universities in various aspects, and projects on digital economy related issues should be strongly supported. Provinces located in the eastern economic zone should continuously improve the tertiary industry and the capacity of industrial and digital integration based on the current situation of digital integration application. So as to improve the quality of the development of digital economy. For the northeast and west inland economic zones. On the premise of paying attention to the integration effect of digital technology with manufacturing and agricultural land, the application degree in service industry should be gradually improved.

4. Strengthen the peripheral environment for the development of digital economy and encourage provinces to increase their openness to the outside world. The peripheral environment of digital economy development includes governance environment, policy guarantee environment and economic development environment. It is the external condition to ensure the maximum effect of digital economy, the important ballast stone for the safety of digital economy environment and the urgent need for the healthy and sustainable development of digital economy. For provinces and cities with poor peripheral environment for the development of digital economy, such as Heilongjiang, Guangxi, Hainan, Yunnan, Tibet, Gansu and other provinces and cities in the northeast and western economic zones, the local government should increase the degree of openness to the outside world and the governance of digital economy environment. Through the establishment of mature talent introduction and enterprise settlement preferential policies to create a stable and good economic development environment. Thus leading the development level of digital economy gradually improved. For Beijing, Shanghai, Jiangsu, Guangdong, Zhejiang and other developed provinces and cities with high level of digital economy development, emphasis should be placed on optimizing the governance environment of digital economy and protecting the environment with policies, so as to stabilize the achievements of digital economy development.

### 5.3 Contribution to research

The innovation of this paper is divided into two points: (1) This paper is based on the collection, collation and research of existing literature, combined with the development process of digital economy. The comprehensive evaluation index system of digital economy is preliminarily constructed from four aspects: digital innovation input, digital infrastructure construction, national economic environment and policy guarantee, and digital integration and application. The grey dynamic clustering and rough intensive reduction theory are used to screen the related indexes, so as to establish a scientific and systematic evaluation index system. (2) The subjective weight of the index is calculated by group FAHP, the objective weight of the index is obtained by using entropy method and improved CRITIC method, and the subjective and objective weight is integrated by using a method based on variance maximization. Finally, according to the improved VIKOR decision model, the digital economy development level of 31 provinces and cities in China from 2013 to 2019 was comprehensively evaluated. It strengthens the reliability, rationality and validity of the empirical analysis conclusion from both theoretical and practical aspects.

### 5.4 Research gaps and direction of further studies

Due to the limited space of this paper, there are still the following shortcomings: (1) The academic community has not reached consensus on the measurement of digital economy at present. Therefore, based on literature research, this paper selects indicators manually under the condition of the availability of indicator data, and selects indicators as completely as possible. According to the development process and characteristics of digital economy, the index system is constructed, but there are still incomplete problems in the index system; (2) Whether the research results of this paper can be extended to the evaluation of digital economy in regional or international areas with significant characteristics. The above problems need to be solved in our follow-up research.

## Supporting information

S1 Appendix (DOCX)
