# Supplementary material for: Evaluation of digital economy development level based on multi-attribute decision theory
Source: PLoS One. 2022 Oct 20;17(10):e0270859. doi: 10.1371/journal.pone.0270859 (PMC9584499; doi:10.1371/journal.pone.0270859)
Supplement: S1 Appendix — (DOCX) [file pone.0270859.s001.docx]

APPENDIX（funding）:

(a)Major Theoretical and Practical Issues in Philosophy and Social Sciences of Shaanxi Province general Project Later funded project "Research on Cultivating Mechanism and Countermeasures of Shaanxi Province Data Factor Market".

(b)General project of Xi 'an Soft Science Research "Research on the Mechanism of digital Economy promoting the High-quality Development of Xi 'an Economy under the Internal circulation Mechanism" (2021-0019).

(c)Key scientific Research Project of Education Department of Shaanxi Province "Targeted Poverty Alleviation Mechanism promoted by New Kinetic Energy of E-commerce" (No.18JZ052).
